# Assessing joint commitment as a process in great apes

**DOI:** 10.1016/j.isci.2021.102872

**Published:** 2021-08-11

**Authors:** Raphaela Heesen, Adrian Bangerter, Klaus Zuberbühler, Katia Iglesias, Christof Neumann, Aude Pajot, Laura Perrenoud, Jean-Pascal Guéry, Federico Rossano, Emilie Genty

**Affiliations:** 1 Institute of Work and Organizational Psychology, University of Neuchâtel, Switzerland; 2 Department of Psychology, Durham University, UK; 3 School of Psychology and Neuroscience, University of St Andrews, Scotland, UK; 4 Institute of Biology, University of Neuchatel, Switzerland; 5 School of Health Sciences (HEdS-FR), HES-SO University of Applied Sciences and Arts of Western Switzerland, Fribourg, Switzerland; 6 German Primate Center (DPZ), Leibniz Institute for Primate Research, Göttingen, Germany; 7 Zoological Park La Vallée des Singes, France; 8 Department of Cognitive Science, University of California San Diego, USA

**Keywords:** Biological sciences, Ethology, Behavioral neuroscience

## Abstract

Many social animals interact jointly, but only humans experience a specific sense of obligation toward their co-participants, a *joint commitment*. However, joint commitment is not only a mental state but also a *process* that reveals itself in the coordination efforts deployed during entry and exit phases of joint action. Here, we investigated the presence and duration of such phases in *N* = 1,242 natural play and grooming interactions of captive chimpanzees and bonobos. The apes frequently exchanged mutual gaze and communicative signals prior to and after engaging in joint activities with conspecifics, demonstrating entry and exit phases comparable to those of human joint activities. Although rank effects were less clear, phases in bonobos were more moderated by friendship compared to phases in chimpanzees, suggesting bonobos were more likely to reflect patterns analogous to human “face management”. This suggests that joint commitment as process was already present in our last common ancestor with *Pan*.

## Highlights

• Great apes exchange signals and gaze before entering and exiting joint actions
• Joint action structure of both ape species resembles that of humans
• Coordinated joint action phases indicate an underlying joint commitment
• Social bonds affect joint action structure more in bonobos than in chimpanzees

## Graphical abstract

**Figure:**
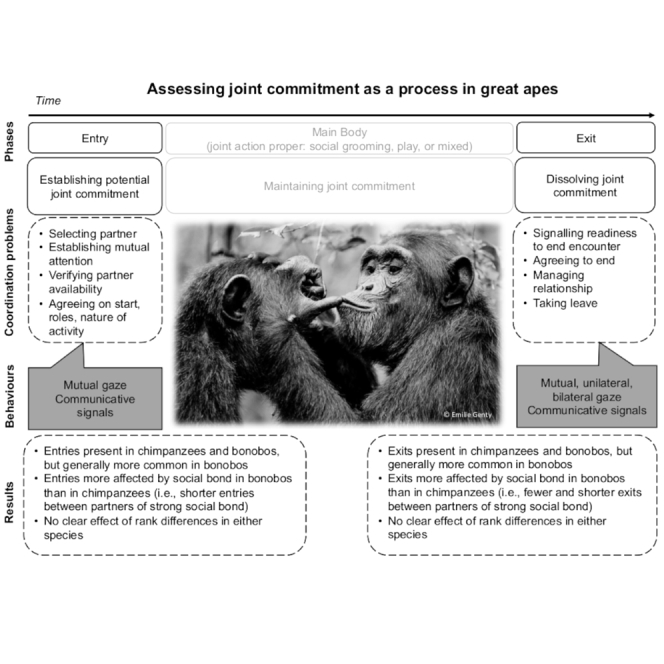

## Introduction

Many social animal species engage in cooperative activities, which sometimes require participants to coordinate joint actions (Boesch, 2002; Holekamp et al., 2007; MacNulty et al., 2014; Pitman and Durban, 2012; Vail et al., 2013). Yet, the evidence as to whether the socio-cognitive abilities underpinning joint actions in animals are comparable to those of humans still remains equivocal (Tomasello and Moll, 2010). In humans, participants in joint actions perform and coordinate their individual actions to make them fit together as part of a whole (Clark, 1996). Successful coordination of joint action relies on predictive inferences about the behavior of other participants. This is fueled by the establishment, maintenance and dissolution of joint commitments (Clark, 2006). Joint commitments entail high-level abilities for cooperation, notably (1) the ability to conceive one's co-participants as intentional agents whose behavior is goal-directed, but also (2) the ability and proclivity to share experiences. While the former is possessed by nonhuman species like great apes (Buttelmann et al., 2012; Call et al., 2004), the latter seems unique to humans (Buttelmann et al., 2017; but see Krupenye et al., 2016; Moore, 2020; Rakoczy, 2015; Tomasello, 2016). Additionally, joint commitments involve a sense of reciprocal obligation to one's partner. This feeling of obligation presumably develops early in human children (Gräfenhain et al., 2009; Gräfenhain et al., 2013), but is disputed in great apes (Warneken et al., 2006; but see new evidence Heesen et al., 2020).

In humans, joint commitments can be viewed as both a *product* and a *process* (Gilbert, 2017). Joint commitment as product refers to the feeling of obligation to fulfill a commitment that has been established. It is visible in humans' reluctance to unilaterally abandon joint endeavors they have committed themselves to, or in attempts to re-engage partners who interrupt their participation. When joint commitments are reneged on, humans feel unjustly treated, triggering emotions that can range from mild irritation (when a friend fails to turn up for a lunch date) to major turmoil (when a fiancé fails to turn up at the altar).

Joint commitment as process refers to the exchange of signals necessary for would-be co-participants to arrive at the mutual belief that they are committed to a course of action where each has his or her part to play. For any spontaneous joint action to emerge, participants need to coordinate on who the participants will be, what roles they will adopt, what actions they will perform, and when and where they will perform them (Clark, 2006). But before they do that, they need to coordinate the very possibility of interacting together, creating the participation framework for a social encounter (Goffman, 1981a; Mondada, 2009). And once the action has been completed, they need to coordinate on gracefully disengaging from the social encounter (Albert and Kessler, 1976; Schegloff and Sacks, 1973). As a result of these coordination processes, spontaneous joint action in humans unfolds into three publicly recognizable phases: the opening or entry phase (establishing joint commitments), the main body (performing the action proper) and closing or exit phase (disengaging from the encounter) (Clark, 1996).

The entry phase comprises all coordinative actions necessary to enter into an interaction. This will typically involve mutual recognition and identification, which often involves greeting rituals (Pillet-Shore, 2012, 2018a, 2018b). Depending on the physical circumstances of the encounter, participants may look at each other while physically far removed, and the mutually visible movement toward one another is informative (Kendon, 1990). At close range, participants build up participation frameworks (Goffman, 1981b; Kendon, 1976), establishing a joint focus of attention and mutual orientation to each other. Participants then establish the nature and content of their encounter, as well as where and when it should take place (Goffman, 1981b; Kendon, 1976, 1990; Mondada, 2009). The joint action transitions from the entry to the main body when the actual business of the encounter is initiated, e.g., taking the first step of a waltz, proceeding to the first topic on the agenda of a meeting, or taking the first bite of a shared meal.

The exit phase comprises all the coordinative actions necessary to get out of a social encounter once the main business of the encounter has been completed. Participants first need to reach the mutual conviction that they are ready to end the encounter (Schegloff and Sacks, 1973). To avoid creating the impression of unilateral disengagement, explicit suggestions to end are often avoided. In English, reciprocal exchanges of *okay* conventionally signal such readiness. When ending an encounter is openly suggested, this is often justified by appealing to externally driven necessities (e.g., “I really have to go”) rather than internal factors (e.g., the desire to end the encounter) (Albert and Kessler, 1976). Once participants agree on their readiness to end the encounter, they may express pleasure at having shared each other's company, project to future encounters (e.g., “see you next week”), and finally take leave of their partners (via expressions of well-wishing like “goodbye”) (Albert and Kessler, 1976; Broth and Mondada, 2013; Clark and French, 1981; Schegloff and Sacks, 1973) before physically disengaging (e.g., walking away).

Besides linguistic expressions, humans also display nonverbal behaviors that delineate each phase (Mondada, 2014). For example, in the entry phase, participants orient their bodies toward each other, gaze at each other and display the intention to touch, hug or kiss each other even before they start talking (Kendon, 1990; Mondada, 2009; Pillet-Shore, 2018a). In the exit phase, participants display the intention to leave by turning their body away from their partner, thus publicly announcing the upcoming end of the interaction, which remains negotiable until officially agreed upon (Broth and Mondada, 2013). Mutual gaze is particularly important to regulate entering into an interaction by displays of availability (Kendon, 1990; Rossano, 2013a), switching turns between speaker- and listener in the main body, as well as ending an encounter (Kendon, 1967). Mutual gaze is a way of monitoring each other but also a means of eliciting specific behavioral responses as evidence of continued engagement in the activity (e.g., to elicit nodding or verbal feedback during a telling a speaker might look toward the addressee at specific moments of a story, see Bavelas et al., 2002; Goodwin, 1984) or simply to mobilize a necessary response to complete an activity (e.g. a speaker might look at a recipient to pressure for an answer and if they engage in mutual gaze this will likely be produced, see Rossano et al., 2009; Stivers and Rossano, 2010). Mutual gaze thus functions as communicative feedback to coordinate the start and end of interaction sequences. Moreover, while sustained gaze by one participant at the presumed end of an interaction sequence would denote the intention to continue, the withdrawal of gaze at that time would display the intention of possibly ending an interaction sequence (Rossano, 2013a).

The coordinative actions that constitute the process of establishing and dissolving joint commitments both reflect and reconstruct the relationship between individual participants. As such, the nature of the relationship is visible in the performance of those coordinative actions. Like any kind of social act, coordinative actions in openings and closings carry the potential for threatening partners' face (honor, reputation, stature), and improperly performed opening and closing phases may result in misunderstandings or affronts. Thus, in humans, these actions will be calibrated to key dimensions of the social relationship via acts of politeness (Brown and Levinson, 1987). Politeness typically involves efforts to reduce the threat of an action to an interlocutor's face. Politeness can involve strategies like indirectness, emphasizing the closeness of a relationship, or minimizing an imposition. Two key dimensions are social distance (how close the individuals are to each other) and power difference (the relative amount of power of one individual over the other) (Brown and Levinson, 1987). Social distance is visible, for example, in parameters of greetings like terms of address or physical contact. In closings, strangers produce more external justifications, more well-wishing statements, and also more statements of positive affect than do friends (Albert and Kessler, 1978), and friends produce less head-nodding and more looking away than do strangers (O'Leary and Gallois, 1985). Power difference is visible, for example, in the amount of effort and respect required to recruit individuals of higher stature for joint actions (Morand, 1996, 2000) or even to approach them (until 1873, in Thailand, approaching the king in court could only be done by crawling on hands and knees, on pain of committing *lèse-majesté*). In other words, people are politer when interacting with higher status and unfamiliar individuals, compared with when interacting with lower status and familiar individuals. So, for example, more efforts are required if a lower status individual (e.g., a peon) recruits a higher status individual (e.g., a king) than the other way around. Additionally, more politeness efforts are required if an individual recruits a socially distant partner (e.g., who they have never met before), as opposed to a familiar partner (e.g., someone living in the same village).

The distinction between joint commitment as process and joint commitment as product opens up new avenues for the assessment of the extent to which joint commitments exist in species other than humans. Indeed, current demonstrations have focused exclusively on joint commitment as product (Gräfenhain et al., 2013; Kachel and Tomasello, 2019; MacLean and Hare, 2013; Pika and Zuberbühler, 2008; Warneken et al., 2006). However, joint commitment as process is logically prior to joint commitment as product: Joint commitments need to be established as common ground between participants before their normative consequences come into play. Joint commitment as process is also ontogenetically prior to joint commitment as product. Indeed, human children learn to interact with others before they develop a sense of the normative dimensions of social conduct (Hamann et al., 2012). The goal of this article is to assess whether joint commitment as process is also *phylogenetically* prior to joint commitment as product. That is, even if humans are arguably the only species where cooperation is imbued with social norms, the processes of getting into and out of social encounters may be observable in other primate species, by assessing the prevalence and components of opening and closing phases bracketing social encounters. Unlike joint commitment as product, which is a binary measure, joint commitment as process offers a potential for continuous assessment because of the many parameters of the opening and closing processes (Genty et al., 2020; Heesen et al., 2017). Opening and closing phases of social encounters thus constitute a potential yardstick for gauging the emergence of precursors of human forms of joint commitment. In particular, if opening and closing phase parameters in other species depend on social distance and power difference between individuals (Brown and Levinson, 1987), this would constitute a striking similarity to human behavior.

The aim of this study was thus to explore joint commitment as a process in chimpanzees and bonobos, by documenting how members of both species get into and out of naturally occurring interactions with conspecifics. We focused on these two species for several reasons. Being our closest relatives, bonobos and chimpanzees represent pivotal models for the reconstruction of the cognitive abilities of our last common ancestor (Prüfer et al., 2012). They can also offer interesting species comparisons for studying social cognition due to differences in their social structure. Both species live in large and complex societies, in which they experience many social challenges, like managing intra-group aggression (Kaburu et al., 2013), gaining access to food and reproductive partners (Surbeck et al., 2012), and securing support during conflicts (Koyama et al., 2006). Contrary to the more despotic dominance hierarchies in chimpanzees, the dominance style of bonobos is documented to be more egalitarian (Hare et al., 2007; but see Jaeggi et al., 2010). While in chimpanzees, males obtain their ranks mainly by means of physical aggression, males in bonobos obtain dominance ranks through amicable bonds with females and through close mother-son relationships (Surbeck et al., 2017). Bonobos are also playful, even in adulthood (Palagi and Paoli, 2007), and exhibit frequent sexual behaviors, also with same-sex partners, which are decoupled from reproduction (Hohmann and Fruth, 2000). The highly frequent socio-sexual contacts in bonobos promote social tolerance and cooperation (Moscovice et al., 2019), reconcile former opponents after conflicts, regulate competition and promote food sharing (see for a review Gruber and Clay, 2016). Contrary to chimpanzees, bonobos generally have increased levels of social tolerance (Hare et al., 2007), emotional and socio-cognitive skills (HerSrmann et al., 2010; Kano et al., 2015; Kret et al., 2016), prosocial behaviors, even with strangers (Tan and Hare, 2013).

We first investigated the prevalence and duration of entry and exit phases in bonobo and chimpanzee joint actions. These included social play, grooming and mixtures of both. Grooming and play serve as particularly adequate joint actions for this research, as they are both carried out in lengthy bouts, occur daily, and involve role-reversals and ample communication (Genty et al., 2020; Heesen et al., 2017). Play is also highly reciprocal, requires active engagement to delineate it from aggression (Flack et al., 2004) and substantial communication (Fröhlich et al., 2016a, 2016b). Chimpanzees and bonobos use gestures and vocalizations to initiate joint activities (Fröhlich et al., 2016a, 2016b; Luef and Pika, 2017; Rossano, 2013b), suggesting that entry phases are present in these species—as previously been demonstrated by other studies. However, to our knowledge, no study has investigated the communicative signals and behaviors used to coordinate the entire interaction using a systematic framework (but see King, 2004 for a descriptive approach), by which the interaction is considered as a global process with entries and exits. Additionally, previous research has not tested whether the global process of joint action coordination is affected by the social dimensions between partners in similar ways as in humans, and whether there are species differences. This however would provide interesting evidence for the apes' sensitivity toward their interaction partners—a crucial aspect of joint action coordination in humans (Brown and Levinson, 1987).

Therefore, we further investigated whether the communicative efforts that constitute these entry and exit phases increased with partners' social distance (measured by social bond strength as inverse proxy) and power difference (measured by rank difference as proxy), and whether there were species differences in this effect. If the apes were to follow patterns similar to human face management, partners who are socially close or of similar rank should produce fewer and shorter entries and exits compared to partners who are socially or hierarchically distant (Brown and Levinson, 1987). Since bonobos are assumed to be more socially tolerant and egalitarian than chimpanzees (e.g., Hare et al., 2007), we predicted stronger effects of social distance in bonobos and stronger effects of social power (dominance rank) in chimpanzees. If so, bonobos should produce fewer and shorter entry and exit phases compared to chimpanzees when partners' social bond strength increases. Comparatively, chimpanzees should produce fewer and shorter entry and exit phases compared to bonobos when partners' rank difference increases.

As for activity types, we generally predicted that play and grooming both involve risks of escalation into aggression (e.g., rough-and-tumble play, grooming a high ranking individual in sensitive body areas), but play might require faster and more mutual coordinative efforts comparatively to grooming, due to their fast pace, simultaneous actions and rough bodily contact (Pellis and Pellis, 1996). Although grooming a higher-ranking individual, for example, might also incur risks (e.g., Fedurek et al., 2015), play likely requires more coordination because both partners play at the same time, with simultaneous roles, in rapid motion, and fast-paced changes in action (see Heesen et al., 2017 for a review). Grooming is slower and thus provides more time for participants to anticipate the other's actions. Consequently, we predicted that social play, compared to other activity types, will reveal the highest frequency of mutual attention, particularly in exit phases, where joint commitment is especially at stake. To this end, we distinguished between mutual exit types (reciprocal exchange of signals and/or gaze), bilateral exit types (partners gaze at each other but at different times), unilateral exit types (only one partner gazes and/or signals), and signal-only exit types (partner/s produce signals but without gazing at each other). Given that bonobos presumably are more egalitarian than chimpanzees, we also predicted that bonobos would generally be more sensitive to joint commitment and manifest more coordination efforts (e.g., mutual eye contact Harrod et al., 2020) especially during the exit phase, i.e., be more likely to engage in mutual exits compared to chimpanzees.

## Results

### General patterns of entry and exit phases in the social interactions of great apes

When entering joint activities, we found that bonobos were more likely to produce entries (i.e., engage in mutual gazes and produce communicative signals) than chimpanzees (Table 1). In bonobos, 90% of interactions contained entries (*N* = 410), whereas in chimpanzees, only 69% of interactions contained entries (*N* = 805). The mean duration of entries was similar in both species, i.e., 12.7 s (*SD* = 20.2 s) in bonobos and 11.5 s (*SD* = 21.0 s) in chimpanzees (Table S1).

**Table 1:** Frequency of joint action beginnings and endings and entry and exit phases per species and activity type

Observations	Bonobos				Chimpanzees				Total
	PL	GR	MX	Subtotal	PL	GR	MX	Subtotal	
Beginnings total	156	227	27	410	151	609	45	805	1,215
Without entries	12	29	0	41	27	222	4	253	294
With entries	144	198	27	369	124	387	41	552	921
Endings total	153	223	21	397	152	602	44	798	1,195
Without exits	7	20	4	31	16	87	8	111	142
With exits	146	203	17	366	136	515	36	687	1,053

PL, play; GR, grooming; MX, mixed activities.

When exiting joint activities, bonobos were also more likely to produce exits (i.e., engage in unilateral/bilateral/mutual gaze and/or produce communicative signals) than chimpanzees (Table 1). In bonobos, 92% of interactions contained exits (*N* = 397) whereas in chimpanzees, only 86% of interactions did (*N* = 798). The mean duration of exits was again similar in both species, i.e., 16.8 s (*SD* = 22.3 s) in bonobos and 13.6 s (*SD* = 14.6 s) in chimpanzees (Table S1). Examples of entry and exit phases can be found in Video S1. Grooming interaction between bonobos of average social bond and large rank difference, with entry phase present, Video S2. Grooming interaction between bonobos of strong social bond and equal rank, with entry phase present, Video S3. Grooming interaction between chimpanzees of weak social bond and large rank difference, with entry phase not present, Video S4. Grooming interaction between chimpanzees of strong social bond and large rank difference, with entry phase present, Video S5. Grooming interaction between bonobos of weak social bond and large rank difference, with exit phase present, Video S6. Play interaction between bonobos of strong social bond and large rank difference, with exit phase not present, Video S7. Play interaction between chimpanzees of weak social bond and large rank difference, with exit phase present, Video S8. Grooming interaction between chimpanzees of strong social bond and large rank difference, with exit phase present, Video S9. Grooming interaction between chimpanzees of strong social bond and small rank difference, with exit phase present, Video S10. Play interaction between bonobos of strong social bond and small rank difference, with exit phase present.

### Effects of social bond strength on the presence and duration of entries and exits

**Figure 1:**
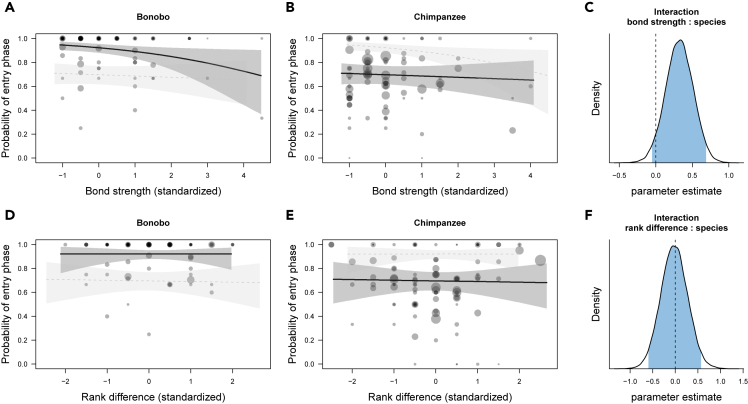
Variation in the presence of entry phases (model 1) (A–C) (A) and (B) portray the relationship between social bond strength (DSI) and the occurrence of entries, separated by species, and (C) shows the posterior distribution of the interaction term between species and bond strength. Each circle represents the proportion of events with entries present out of all events. (D–F) (D) and (E) show the relationship between rank difference and the occurrence of entries, and (F) shows the posterior distribution of the interaction term between species and rank difference. Values for DSI and rank difference were binned with a width of 0.5. Circle size is proportional to the total number of events. Circles are colored with transparent filling and hence overlap (multiple individuals with the same value will appear darker). The lines represent model fits along with shaded 95% credible bands. To aid comparison between species, in each panel we added the model results for the other species in fainter color and with dashed lines. In the posterior distributions, the blue area corresponds to the 95% credible interval of the estimated effect with the value of 0 highlighted by a vertical dashed line.

For the presence of entries, we found a weak interaction effect between species and social bond strength (dyadic composite sociality index [DSI]) (Figures 1A–1C; b = 0.33; SD = 0.19; 95% CrI [−0.05, 0.70]; Table S2 model 1), with bonobos being slightly less likely to produce entries when interacting with socially close than distant partners, compared to chimpanzees. Bonobos were also substantially more likely to produce shorter entries when interacting with socially close than distant partners, while the duration of chimpanzee entries was unaffected by social bond strength (Figures 3A–3C; b = 0.17; SD = 0.08; 95% CrI [0.01, 0.33]; Table S2 model 2).

**Figure 2:**
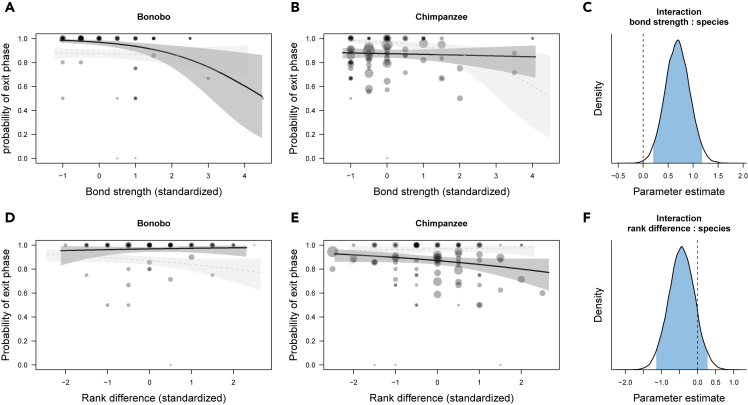
Variation in the presence of exit phases (model 3) (A) and (B) portray the relationship between social bond strength (DSI) and the occurrence of exits, separated by species, and (C) shows the posterior distribution of the interaction term between species and bond strength. Each circle represents the proportion of events with exits present out of all events. (D) and (E) show the relationship between rank difference and the occurrence of exits, and (F) shows the posterior distribution of the interaction term between species and rank difference. Values for bond strength and rank difference were binned with a width of 0.5. Circle size is proportional to the total number of events. Circles are colored with transparent filling and hence overlap (multiple individuals with the same value will appear darker). The lines represent model fits along with shaded 95% credible bands. To aid comparison between species, in each panel we added the model results for the other species in fainter color and with dashed lines. In the posterior distributions, the blue area corresponds to the 95% credible interval of the estimated effect with the value of 0 highlighted by a vertical dashed line.

As for exit phases, bonobos were generally more likely to produce exits compared with chimpanzees (Figure 2), but again less likely to produce exits with socially close partners compared with distant partners (Figures 2A–2C; b = 0.69; SD = 0.25, 95% CrI [0.20, 1.19]; Table S2 model 3). In contrast, the probability to produce exits was unaffected by social bond strength in chimpanzees. Similar to patterns in entries, bonobos also produced shorter exits when social bond strength increased compared with chimpanzees (Figures 4A–4C; b = 0.27; SD = 0.07, 95% CrI [0.13, 0.42]), Table S2 model 4).

### Effects of rank difference on the presence and duration of entries and exits

**Figure 3:**
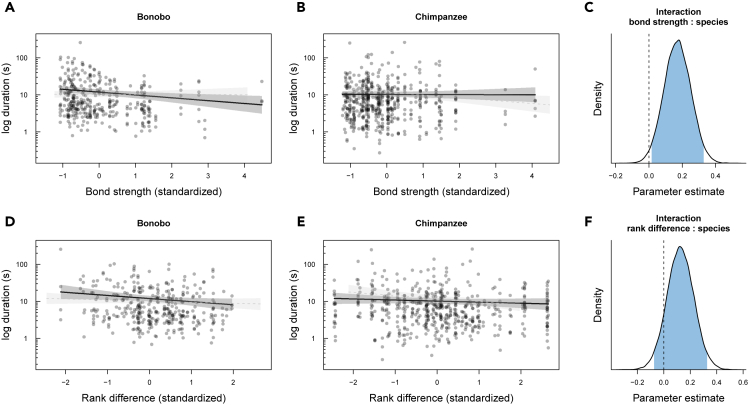
Variation in the log duration of entry phases (model 2) (A–C) (A) and (B) depict the relationship between social bond strength (DSI) and the log duration of entries, separated by species, and (C) shows the posterior distribution of the interaction term between species and bond strength. (D–F) (D) and (E) show the relationship between rank difference and the log duration of entries, and (F) reveals the posterior distribution of the interaction term between species and bond strength. Each circle represents the log duration of entry events depending on the partners' bond strength or rank difference. Values for DSI and rank difference were binned with a width of 0.5. Circles are colored with transparent filling and hence overlap (multiple individuals with the same value) will appear darker. The lines represent model fits along with shaded 95% credible bands. To aid comparison between species, in each panel we added the model results for the other species in fainter color and with dashed lines. In the posterior distributions, the blue area corresponds to the 95% credible interval of the estimated effect with the value of 0 highlighted by a vertical dashed line.

We found no effect of rank difference on the presence of entry phases in either species (Figures 1D–1F; b = −0.02; SD = 0.30, 95% CrI [−0.61, 0.57]; Table S2 model 1). Although, for the duration of entry phases, the interaction between species and rank difference was not substantial (Figure 3F), there was a tendency of bonobos to produce slightly shorter entries when initiating interactions with lower than with higher-ranking partners, and such a tendency was not apparent in chimpanzees (Figures 3D–3F; b = 0.13; SD = 0.10, 95% CrI [−0.07, 0.33]; Table S2 model 2).

**Figure 4:**
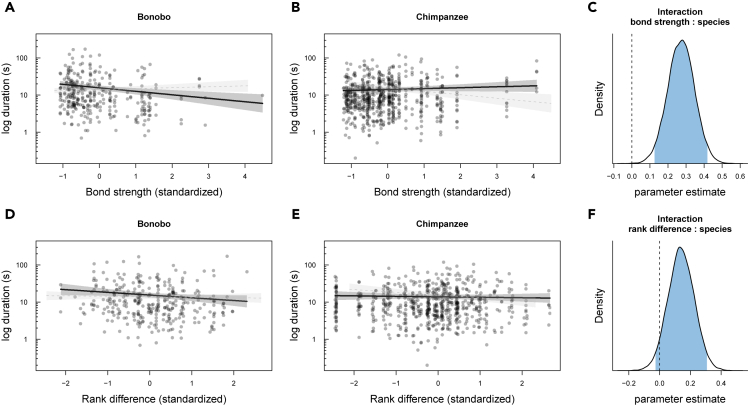
Variation in the log duration of exit phases (model 4) (A–C) (A) and (B) depict the relationship between social bond strength (DSI) and the log duration of exits, separated by species, and (C) shows the posterior distribution of the interaction term between species and bond strength. (D–F) (D) and (E) show the relationship between rank difference and the log duration of exits, and (F) reveals the posterior distribution of the interaction term between species and bond strength. Each circle represents the log duration of exit events depending on the partners' bond strength or rank difference. Values for DSI and rank difference were binned with a width of 0.5. Circles are colored with transparent filling and hence overlap (multiple individuals with the same value) will appear darker. The lines represent model fits along with shaded 95% credible bands. To aid comparison between species, in each panel we added the model results for the other species in fainter color and with dashed lines. In the posterior distributions, the blue area corresponds to the 95% credible interval of the estimated effect with the value of 0 highlighted by a vertical dashed line.

Likewise, there were no clear interactions between species and rank difference in duration or presence of exit phases (Figures 2F and 4F). For exit presence, there was only a slight tendency of chimpanzees (compared to bonobos, Figure 2D) to produce fewer exit phases when they were higher-ranking than their interaction partners (Figure 2E; b = −0.44; SD = 0.36; 95% CrI [−1.15, 0.28]; Table S2 model 3). For exit duration, there was a weak tendency of bonobos to produce shorter exits when terminating interactions with lower than with higher-ranking partners (Figure 4D), and this tendency was not apparent in chimpanzees (Figures 4E and 4F; b = 0.14; SD = 0.09; 95% CrI [−0.03, 0.31]); Table S2 model 4).

### Effects of species and activity type on the presence of mutual exits

We found that bonobos engaged in mutual exits in 51% of interactions (bilateral: 20%, unilateral: 29%, signals-only: 0%; *N* = 324; Table S3), whereas chimpanzees engaged in mutual exits in only 39% of interactions (bilateral: 21%, unilateral: 39%, signals-only without gaze: 1%; *N* = 659; Table S3). Although the species difference was weak, model 5 (Table S2) revealed that chimpanzees tended to produce fewer mutual exits than bonobos (Figure S1A; b = −0.33, SD = 0.19, 95% CrI [−0.71, 0.04]).

As predicted, we also found a substantial effect of activity type on the presence of mutual exits (Figure S1B; Table S2 model 5). Across species, mutual exits were most common in social play (60%, *N* = 268 exits), followed by mixed interactions (53%, *N* = 49 exits) and grooming (35%, *N* = 666 exits). Model 5 confirmed this, estimating that grooming interactions terminated with fewer mutual exits than play interactions (Figure S1B; b = −1.00; SD = 0.17; 95% CrI [−1.35, −0.67]). On the contrary, however, there was no difference in the use of mutual exits between play and mixed interactions (Figure S1B; b = −0.28; SD = 0.33, 95% CrI [−0.93, 0.36]).

## Discussion

When humans intend to carry out a joint action, such as to move a large table, they do this by first establishing a joint commitment with their partners and, once the action is completed, by terminating their commitment. Joint actions, in other words, are encased by joint commitment, a process that starts before and terminates after the actual action. We tested the hypothesis that joint commitment *as process* is evolutionarily old and also present in great apes. To do so, we investigated two main joint actions commonly carried out by great apes, social grooming and play, by collecting and analyzing a large number of initiations and terminations. Our data showed that bonobos and chimpanzees consistently exchanged mutual gaze and communicative signals *prior to engaging* in these joint actions. When exiting joint actions, we found the same pattern insofar as subjects did not just unilaterally abandon a joint action but engaged with their partners via gaze and/or communicative signals also *before leaving*. Particularly in bonobos, this process was moderated by variables related to their relationships with some similarities to what in humans is considered “social etiquette” or “politeness” (Brown and Levinson, 1987).

Joint commitment is the “glue” that holds joint actions together (Clark, 2006), and it is often viewed as a static state of mutual obligation. Allegedly, only humans have the cognitive capacity to experience joint commitment *as product* (Tomasello and Carpenter, 2007; Warneken et al., 2006)—although new evidence from bonobos may require a revision of this claim (Heesen et al., 2020). We here argued that joint commitment is not just a product but also a process (Genty et al., 2020; Heesen et al., 2017). In humans, this process is visible through interactive sequences during which participants gradually enter a state of mutual obligation, and then gradually exit that state without harming their social relations (Bangerter et al., 2010; Chevalley and Bangerter, 2010; Clark, 2006). Joint commitment, thus, is both process and product and can be studied in both ways in our own and other species (e.g., see Heesen et al., 2020).

Here, we were interested in the evolutionary origins of joint commitment as a process, by asking whether great ape joint action is accompanied by specific entry and exit phases. Our findings suggested that this is the case, pointing to a shared ancestry and phylogenetic continuity of joint commitment *as process.* However, we also found relevant species differences, especially also within the great apes. In particular, bonobos had more prevalent entry and exit phases than chimpanzees, their exits contained more mutual gazes and gaze-tactile gesture combinations (“mutual exits”, Figure S1), and their phase structure was consistently more sensitive to social bond strength compared to those of chimpanzees. Chimpanzees, in contrast, were less sensitive to the social bonds between themselves and their partners, which generally fits with previous notions of socio-cognitive differences between the two species (Hare et al., 2007; Herrmann et al., 2010). Along the same lines, we also predicted that, due to the chimpanzees' more despotic dominance relations, chimpanzees would more likely be affected by rank differences than bonobos, yet this prediction was not clearly reflected by our data. Although chimpanzees tended to produce *fewer* exit phases when they themselves were higher-ranking than their partners, bonobos in turn tended to produce slightly *shorter* entries and exits when they themselves were higher-ranking than their partners.

These findings give rise to a number of conclusions concerning joint action coordination in great apes. First, bonobos generally follow a pattern that resembles human face management more so than that of chimpanzees, especially with respect to social bonds. For example, Figures 1, 2, 3, and 4 (A-C) showed that bonobos—more so than chimpanzees—produced fewer and shorter entry and exit phases as the partners' social bond strength increased. These patterns are analogous to the relational parameters that govern the management of “face” in human social interactions. Yet, although this analogy is striking, we are not suggesting that great apes are aware of, or care about each other's face in the sense that humans do; it is more likely that these patterns reflect higher arousal and alertness when interacting with higher-ranking and more socially distant individuals. Humans tend to increase their coordination efforts (via politeness strategies) when interacting with unfamiliar individuals (Brown and Levinson, 1987). However, although bonobos overall seemed more affected than chimpanzees by both of the social dimensions than chimpanzees, it is to note that rank differences appeared to be less important than social bonds. This finding is not surprising and fits well with the documented differences in the two species' dominance styles and prosocial tendencies. Bonobos are generally perceived as more prosocial, more tolerant and less despotic than chimpanzees (Hare et al., 2007; but see Jaeggi et al., 2010; Tan et al., 2017). Less despotic societies allow for more negotiation between partners, potentially fostering the emergence of more complex communication systems (Freeberg et al., 2012). The fact that bonobos responded more to social bond strength also suggests that bonobos, more so than chimpanzees, may take into account interactional histories and emotional bonds with certain individuals—a possible precursor for the development of dyad-specific conventions (Tomasello, 2016).

Patterns of evolutionary change are determined both by phylogenetic inertia and species-specific local adaptations. While the close phylogenetic proximity between bonobos, chimpanzees, and humans predicts similarities in joint commitment processes, our data suggest that differences in the socio-ecology of bonobos and chimpanzees have led to differences in the prevalence of joint commitments. To further disentangle these species differences, future studies will be needed to investigate other aspects of joint commitment, such as the types of signals used across phases and across social relations. To address the wider phylogenetic history, future studies should also assess joint action coordination in other great ape species, as well as in more distantly related primate and non-primate species.

Because the process of establishing, maintaining and dissolving joint commitments is multidimensional, it provides a yardstick for the comparative assessment of the evolution of joint action coordination capabilities not only in great apes but also in other species. By applying a joint action framework developed for human interactions, we were able to document how apes get into and out of joint actions, and whether there are levels of partner-awareness comparable to those of humans. Our findings suggest these parallels exist, which offers a new paradigm for studying joint commitment in an ecologically valid setting. This is in contrast to the current literature that is largely biased toward laboratory experiments often with ecologically irrelevant activities (Warneken et al., 2006). Although our data cannot address the cognitive complexity underlying the observed joint action coordination patterns, other research has additionally shown that great apes track the progress of joint actions as well as the performance of their partners, as evidenced by their choices of interaction partners (Melis et al., 2006).

How aware are great apes of their partners during joint actions? The basic component constituting entry phases in chimpanzees, bonobos and humans is mutual gaze, but this has also been demonstrated in other species, such as marmosets before a joint experimental task (Miss and Burkart, 2018), suggesting that mutual gaze is widespread in social animals. Depending on the species, but also on environmental contingencies, additional components might include visible approach/retreat, activity-specific gestures or body positioning (Fröhlich et al., 2016a, 2016b), vocalizations (Luef and Pika, 2017), and linguistic communication in humans (Clark, 1996). Regarding exits, however, we are not aware of any previous systematic animal studies demonstrating mutual gaze and accompanying signaling behavior. By documenting how two species of great apes got into and out of joint action, we were able to show that both chimpanzees and bonobos—at several study sites—have specific entry and exit phases (Fröhlich et al., 2016a, 2016b; see also Rossano, 2013b). Since the length and duration of exit phases—particularly in bonobos—were more strongly affected by social variables than entry phases, patterns analogous to human face management might generally be more represented in the exit phase of great ape joint activities. Future studies attempting to identify such patterns beyond humans using a comparative approach might devote specific attention to the *termination* of joint activities. Also relevant is whether great apes re-engage each other after interruptions of joint action — a gold standard for joint commitment, something that has recently been demonstrated in related research (Heesen et al., 2020).

In conclusion, our findings show that two species of great apes habitually go through the same process and stages as humans when establishing, executing and terminating joint actions. They deploy mutual gaze during entry phases and before transitioning into the main phases of joint actions and termination is not abrupt but embedded into a distinct exit phase, often instigated by mutual gaze. These parallels suggest that the phylogenetic roots of joint action, along with the capacity to establish joint commitment as process, might be phylogenetically older than previously assumed (Tomasello, 2016), having emerged prior to—or with—the common ancestor of *Homo* and *Pan*.

### Limitations of the study

Our observations focus on great apes housed in captivity. Hence, it is reasonable to ask whether our observations would generalize to social interactions in the wild. Although we focused on spontaneous (natural) behaviors, captive individuals live in unnaturally close proximity to each other, with increased terrestriality and visibility, which may trigger more complex social and communicative strategies compared to free-ranging individuals (Fröhlich and van Schaik, 2018). In addition, the spare time allowed by food provisioning, combined with environmental enrichment, might further stimulate enhanced social strategies and more complex communicative repertoires (Kummer and Goodall, 1985). Thus, research on joint commitment as process in wild populations would be required to address questions regarding the evolutionary origins of joint commitment. Second, although our data show that joint commitment processes in great apes resemble those of humans, it remains unclear whether great apes also experience a corresponding underlying mental state of shared-intentionality (Tomasello and Carpenter, 2007; Tuomela, 2007), or whether this is mediated by the presence of language which may facilitate sharing experiences, thoughts and conventions (Clark, 1996). Here, further research on the communication strategies pursued by apes would be relevant in how they coordinate joint action phases, i.e., how they advertise their intentions to initiate, suspend, resume or terminate a joint action (Genty et al., 2020; Heesen et al., 2020; Heesen et al., 2017). Third, as always it is impossible to draw strong conclusions about evolutionary processes from studying only two species of apes. The patterns described here may also be found in other species, such as social carnivores, and may even be absent in more solitary species, such as orangutans, which would then support hypotheses based on learning or convergent evolution. Comparative research on distantly related species that do or do not regularly engage in joint actions would be especially revealing (Heesen et al., 2017).

## STAR★Methods

### Key resources table

**Table:** 

REAGENT or RESOURCE	SOURCE	IDENTIFIER
**Deposited data**		

Data S1	Repository and manuscript (supplementary information)	https://doi.org/10.6084/m9.figshare.14723493

**Software and algorithms**		

R code	repository	https://doi.org/10.6084/m9.figshare.14723493

**Other**		

Videos S1, S2, S3, S4, S5, S6, S7, S8, S9, and S10	Manuscript (supplementary information)	Videos S1, S2, S3, S4, S5, S6, S7, S8, S9, and S10, Uploaded with supplementary information

### Resource availability

#### Lead contact

Requests for further information, resources and materials should be directed to and will be fulfilled by the lead contact, Dr. Raphaela Heesen (heesenr1@gmail.com).

### Materials availability

Evidence from video recordings analyzed in this study can be found in the supplementary information (Videos S1, S2, S3, S4, S5, S6, S7, S8, S9, and S10).

Video S1. Grooming interaction between bonobos of average social bond and large rank difference, with entry phase present This video exemplifies an entry phase in a social grooming activity between two bonobos. Kelele (male), the phase initiator, approaches Diwani (male) and both mutually gaze at each other (start of the entry phase) before Kelele sits next to Diwani. A sequence of communicative signal exchanges starts. Diwani reaches with his leg (gesture), and then presents his arm two times (gestures), upon which the two remain in the entry phase for a moment until Kelele jerks with his head (gesture) and Diwani again presents his arm to Kelele (gesture). Consequently, Kelele starts grooming Diwani's arm (which was presented to him) and the entry phase stops. The main body of grooming starts; *Movie annotations*: black band with title = indicating the type and duration of the phase; green arrows = location of interaction partners; blue/red double arrows = mutual gaze; red single arrow = unilateral gaze; green circle = communicative signal (e.g., gesture); *Credit:* Raphaela Heesen and Emilie Genty; *Species*: Bonobo; *Site*: La Vallée des Singes, France; *Activity type*: Grooming; *Entry:* Present; *Duration*: 39.7 s; *Dyad ID:* Diwani + Kelele; *Phase initiator:* Kelele; *Social bond (standardized, mean = 0.00):* 0.01 (average); *Rank difference (standardized, mean =* −*0.01)*: −1.02 Elo scores (initiator lower ranking). Related to Figures 1 and Figure 3.

Video S2. Grooming interaction between bonobos of strong social bond and equal rank, with entry phase present This video exemplifies an entry phase in a social grooming activity between two bonobos. David (male), the phase initiator, approaches Daniela (female) and both mutually gaze at each other (start of the entry phase) before Daniela then immediately starts grooming David's back (end of entry phase, start of main body). No communicative signals are exchanged in this entry phase; *Movie annotations*: black band with title = indicating the type and duration of the phase; green arrows = location of interaction partners; blue/red double arrows = mutual gaze; red single arrow = unilateral gaze; green circle = communicative signal (e.g., gesture); *Credit:* Raphaela Heesen and Emilie Genty; *Species*: Bonobo; *Site*: La Vallée des Singes, France; *Activity type*: Grooming; *Entry:* Present; *Duration*: 2.9 s; *Dyad ID:* David + Daniela; *Phase initiator:* David; *Social bond (standardized, mean = 0.00):* 2.25 (strong); *Rank difference (standardized, mean =* −*0.01)*: −0.01 Elo scores (initiator slightly lower ranking). Related to Figures 1 and Figure 3.

Video S3. Grooming interaction between chimpanzees of weak social bond and large rank difference, with entry phase not present This video is an example for an interaction that starts without a present entry phase. Roy (male), the phase initiator, approaches Conan (male). There is no mutual gaze exchange, nor any communicative signal exchange. Once Roy sits close enough to Conan, he immediately starts grooming his body; *Movie annotation*: black band with title = indicating the type and duration of the phase; green arrows = location of interaction partners; blue/red double arrows = mutual gaze; red single arrow = unilateral gaze; green circle = communicative signal (e.g., gesture); *Credit:* Raphaela Heesen and Emilie Genty; *Species*: Chimpanzee; *Site*: La Vallée des Singes, France; *Activity type*: Grooming; *Entry:* Not present; *Duration*: NA; *Dyad ID:* Roy + Conan; *Phase initiator:* NA; *Social bond (standardized, mean = 0.00):* −0.69 (weak); *Rank difference (standardized, mean =* −*0.01)*: 0.98 Elo scores (initiator higher ranking). Related to Figures 1 and Figure 3.

Video S4. Grooming interaction between chimpanzees of strong social bond and large rank difference, with entry phase present This video exemplifies an entry phase in a social grooming activity between two chimpanzees. Madino (male) approaches Macourie (female) and both mutually gaze at each other (start of the entry phase). Macourie then uses a series of gestures, first attempting to grab Madingo, then touching his shoulder and back (gestures), and finally, grab-pulling him at his hips (gesture). Macourie then starts grooming him on his shoulder once he is sitting in close proximity. The entry stops with the first grooming movements, upon which the main body starts; *Movie annotations*: black band with title = indicating the type and duration of the phase; green arrows = location of interaction partners; blue/red double arrows = mutual gaze; red single arrow = unilateral gaze; green circle = communicative signal (e.g., gesture); *Credit:* Raphaela Heesen and Emilie Genty; *Species*: Chimpanzee; *Site*: La Réserve Africaine de Sigean, France; *Activity type*: Grooming; *Entry:* Present; *Duration*: 9.1s; *Dyad ID:* Macourie + Madingo; *Phase initiator:* Macourie; *Social bond (standardized, mean = 0.00):* 1.49 (strong); *Rank difference (standardized, mean =* −*0.01)*: 1.01 Elo scores (initiator higher ranking). Related to Figures 1 and Figure 3.

Video S5. Grooming interaction between bonobos of weak social bond and large rank difference, with exit phase present This video exemplifies an exit phase in a social grooming activity between two bonobos. Mali (female) is grooming Loretta (female) in the main phase. Mali, the phase initiator, then stops grooming Loretta, upon which the exit phase starts. Before she takes leave, Mali leans to the side (into the visual field of Loretta) and the two mutually gaze at each other. Consequently, Mali produces an idiosyncratic gesture (putting her thumb into her mouth) upon which Loretta nods her head (gesture) in a communicative signal exchange. Mali then leaves and lays down behind Loretta (still with the thumb in her mouth) and gazes back at her once more. The exit phase stops and no further interaction follows; *Movie annotations*: black band with title = indicating the type and duration of the phase; green arrows = location of interaction partners; blue/red double arrows = mutual gaze; red single arrow = unilateral gaze; green circle = communicative signal (e.g., gesture); *Credit:* Raphaela Heesen and Emilie Genty; *Species*: Bonobo; *Site*: San Diego Zoo, USA; *Activity type*: Grooming; *Exit:* Present; *Duration*: 15.5s; *Exit Type:* Mutual exit; *Dyad ID:* Mali + Loretta; *Phase initiator:* Mali; *Social bond (standardized, mean = 0.00):* −0.58 (weak); *Rank difference (standardized, mean =* −*0.01)*: −0.57 Elo scores (initiator lower ranking). Related to Figures 2 and Figure 4.

Video S6. Play interaction between bonobos of strong social bond and large rank difference, with exit phase not present This video is an example for an interaction that ends without a present exit phase. Moko (male), the phase initiator, suddenly breaks off the playful chase with Khalessi (female), upon which the two bonobos walk into different directions without gazing at each other nor communicating; *Movie annotations*: black band with title = indicating the type and duration of the phase; green arrows = location of interaction partners; blue/red double arrows = mutual gaze; red single arrow = unilateral gaze; green circle = communicative signal (e.g., gesture); *Credit:* Raphaela Heesen and Emilie Genty; *Species*: Bonobo; *Site*: La Vallée des Singes, France; *Activity type*: Play; *Exit:* Not present; *Duration*: NA; *Exit Type*: NA; *Dyad ID:* Khalessi + Moko; *Phase initiator:* NA; *Social bond (standardized, mean = 0.00):* 1.37 (strong); *Rank difference (standardized, mean =* −*0.01)*: 1.07 Elo scores (initiator higher ranking). Related to Figures 2 and Figure 4.

Video S7. Play interaction between chimpanzees of weak social bond and large rank difference, with exit phase present This video exemplifies an exit phase in a play activity between two chimpanzees. Kume (male) is engaging in chase play with Colebe (male) in the main phase. Colebe, the phase initiator, then stops to run and play chase and turns around, upon which the exit phase starts. The two mutually gaze at each other, Colebe attempts to reinstate the play interaction, but Kume pushes (gesture) his head, upon which Colebe grabs (gesture) the hand by which Kume is holding onto Colebe's head. Kume then drops his hand, the two hold hands for a moment while gazing at each other again, they then perform a head on head (gesture) while facing each other. Colebe then sits down, Kume walks away, Colebe gazes one more time at Kume walking away, and the exit phase stops. No further interaction follows; *Movie annotations*: black band with title = indicating the type and duration of the phase; green arrows = location of interaction partners; blue/red double arrows = mutual gaze; green circle = communicative signal (e.g., gesture); *Credit:* Raphaela Heesen and Emilie Genty; *Species*: Chimpanzee; *Site*: Basel Zoo, Switzerland; *Activity type*: Play; *Exit:* Present; *Duration*: 22.4s; *Exit Type*: Mutual exit; *Dyad ID:* Kume + Colebe; *Phase initiator:* Colebe; *Social bond (standardized, mean = 0.00):* −1.30 (weak); *Rank difference (standardized, mean =* −*0.01)*: −1.30 Elo scores (initiator lower ranking). Related to Figures 2 and Figure 4.

Video S8. Grooming interaction between chimpanzees of strong social bond and large rank difference, with exit phase present This video exemplifies an exit phase in a social grooming activity between two chimpanzees. Macourie (female) is grooming Madingo (male) in the main phase. Macourie, the phase initiator, then stops grooming Madingo, upon which the exit phase starts. Macourie then unilaterally gazes at Madingo and touches his back (gesture) before moving away. The exit phase stops and no further interaction follows; *Movie annotations*: black band with title = indicating the type and duration of the phase; green arrows = location of interaction partners; blue/red double arrows = mutual gaze; red single arrow = unilateral gaze; green circle = communicative signal (e.g., gesture); *Credit:* Raphaela Heesen and Emilie Genty; *Species*: Chimpanzees; *Site*: La Réserve Africaine de Sigean, France; *Activity type*: Grooming; *Exit:* Present; *Duration*: 3.6s; *Exit Type*: Unilateral exit; *Dyad ID:* Macourie + Madingo; *Phase initiator:* Macourie; *Social bond (standardized, mean = 0.00):* 1.49 (strong); *Rank difference (standardized, mean =* −*0.01)*: 1.01 Elo scores (initiator higher ranking). Related to Figures 2 and Figure 4.

Video S9. Grooming interaction between chimpanzees of strong social bond and small rank difference, with exit phase present This video exemplifies an exit phase in a social grooming activity between two chimpanzees. Wonder (male) is grooming Conan (male) in the main phase. Wonder, the phase initiator, then stops grooming Conan's hand, upon which the exit phase starts. Wonder first walks away to the left side on the branch, but then turns around and re-approaches Conan. They mutually gaze at each other and Wonder grabs Conan's hand (Gesture) while touching his hand with his mouth. Wonder then leaves and sits at a different location, while Conan gazes back at him one more time. The exit phase stops and no further interaction follows; *Movie annotations*: black band with title = indicating the type and duration of the phase; green arrows = location of interaction partners; blue/red double arrows = mutual gaze; red single arrow = unilateral gaze; green circle = communicative signal (e.g., gesture); *Credit:* Raphaela Heesen and Emilie Genty; *Species*: Chimpanzees; *Site*: La Vallée des Singes, France; *Activity type*: Grooming; *Exit:* Present; *Duration*: 22.1s; *Exit Type*: Mutual exit; *Dyad ID:* Wonder + Conan; *Phase initiator:* Wonder; *Social bond (standardized, mean = 0.00):* 0.92 (strong); *Rank difference (standardized, mean =* −*0.01)*: 0.44 Elo scores (initiator higher ranking). Related to Figures 2 and Figure 4.

Video S10. Play interaction between bonobos of strong social bond and small rank difference, with exit phase present This video exemplifies an exit phase in a play activity between two bonobos. Belle (female) is engaging in wrestle play with Maddie (female) in the main phase. Belle and Maddie then stop playing due to an interruption of a third party member, after which the exit phase starts. The two separate as Belle walks off, shortly after which she turns around to gaze at Maddie and produces a concave back present (body signal). Following the body signal, both bonobos mutually gaze at each other and cross each other's way, during which Belle puts her hand on (gesture) Maddie's back. The two then separate, Maddie walks off while Belle is gazing back at her once more. Shortly after Belle walks off and the exit phase stops. No further interaction follows; *Movie annotations*: black band with title = indicating the type and duration of the phase; green arrows = location of interaction partners; blue/red double arrows = mutual gaze; red single arrow = unilateral gaze; green circle = communicative signal (e.g., gesture or body signal); *Credit:* Raphaela Heesen and Emilie Genty; *Species*: Bonobo; *Site*: San Diego Zoo, USA; *Activity type*: Play; *Exit:* Present; *Duration*: 9.88s; *Exit Type*: Mutual exit; *Dyad ID:* Maddie + Belle; *Phase initiator:* mutual; *Social bond (standardized, mean = 0.00):* 2.75 (strong); *Rank difference (standardized, mean =* −*0.01)*: −0.25 Elo scores. Related to Figures 2 and Figure 4.

### Data and code availability

• The data supporting the article have been deposited at figshare.com and are publicly available as of the date of publication. DOI can be found in key resource table.
• The R code to recreate the analyses and plots supporting the article have been deposited at figshare.com and are publicly available as of the date of publication. DOI can be found in key resource table.
• Any additional information required to reanalyze the data reported in this paper is available from the lead contact upon request.

### Experimental model and subject details

We collected observational data from two groups of bonobos and three groups of chimpanzees at four zoological sites (total *N* = 50 individuals, see Table S4). RH observed one group of bonobos at San Diego Zoo, USA, from January 2017 to April 2017 (*N* = 9, age range = 4–44 years, 6 females, 3 males), and a second group of bonobos at La Vallée des Singes, France, from April 2017 to September 2017 (*N* = 16, age range = 4–52 years, 10 females, 6 males). In this same time period, a trained research assistant collected data from a group of chimpanzees at La Vallée des Singes, France (*N* = 7, age range = 9–23 years, 3 females, 4 males). LP observed a second group of chimpanzees at Basel Zoo, Switzerland from October 2016 to February 2017 (*N* = 9, age range = 7–49 years, 6 females, 3 males). AP observed a third group of chimpanzees at La Réserve Africaine de Sigean, France, from June to August 2018 (*N* = 9, age range = 5–45 years, 6 females, 3 males).

The apes at La Vallée des Singes are housed in large enclosures consisting of an outdoor island-type enclosure with a forest area and climbing structures in grassy areas (8,000 m^2^ bonobos, 7,500 m^2^ chimpanzees), and an indoor enclosure with enrichment and climbing structures (600 m^2^ bonobos, 220 m^2^ chimpanzees). Food is distributed five to six times a day and includes primate pellets, fruits, and vegetables (and a daily portion of rice for chimpanzees, and a weekly portion of rice for bonobos). The apes are also fed nuts and meat once a week, and eggs twice a week. Both species can also forage in their outdoor enclosure (wild berries, herbaceous vegetation).

The bonobos at the San Diego Zoo are housed in an enclosure including both an outdoor (560 m^2^) and an indoor enclosure (296 m^2^). The outdoor enclosure consists of climbing structures, a fresh-water stream, and elements of enrichment. The group receives abundant food types 3–4 times a day (primate pellets and cereals, fruits, vegetables, nuts), and supplementary feeds several times a week (honey, peanut butter, popcorn, seeds), often provided through enrichment toys.

The chimpanzees at Basel Zoo are housed in a building comprising six lodges in the inside enclosure (total 233 m^2^) and two in the outside enclosure (total 477 m^2^). The lodges are provided with climbing structures, ropes, puzzle boxes and other enrichments, and are connected to each other. The subjects can freely roam between them; two are not visible to visitors. Food is provisioned six times a day, including salad, vegetables, fruits, eggs and primate pellets.

The chimpanzees housed at La Réserve Africaine de Sigean are kept in a 200 m^2^ indoor enclosure and two 1.3 ha outdoor island areas. During the day, they are kept on the outdoor island that features natural vegetation, bushes, trees, sand, a surrounding water pond, climbing structures, bridges and shelters. The group is provided with food six times a day, including vegetables, fruits, primate pellets and nuts, and chicken and eggs once a week.

Fresh water for all groups is always provided *ad libitum,* and all groups (except the chimpanzees from Basel Zoo) can also drink from water sources in their outdoor enclosures.

#### Institutional permission

We received ethical agreement for this study from the Commission d'Ethique de la Recherche of the University of Neuchâtel (agreement number: 01-FS-2017), the internal ethical committee of La Vallée des Singes, the UCSD Human Research Protections Program (Project number: 161452S), the IACUC committee of San Diego Zoo Global (Project number #17-007), the internal ethical committee of the Réserve Africaine de Sigean, and the Kantonales Veterinäramt BS at Basel Zoo.

#### Data collection

Data were collected using continuous focal behavior sampling (Altmann, 1974), maintaining a balanced record of focal samples across individuals. All focal samples (15–30 min, depending on the site and its visibility conditions) were recorded using two Panasonic HC-V770 camcorders with two external Sennheiser unidirectional microphones (MKE 400). Overall, we collected 1298.55 hr of observation (600.25 hr for bonobos, 698.30 hr for chimpanzees, see Table S4), with on average 30.00 hr per bonobo at San Diego Zoo, 20.64 hr (*SD* = 0.52 hr) per bonobo at La Vallée des Singes, 37.01 hr (*SD* = 0.45 hr) per chimpanzee at La Vallée des Singes, 30.00 hr per chimpanzee at Basel Zoo, and 18.08 hr (*SD* = 0.42 hr) per chimpanzee at La Réserve Africaine de Sigean.

To collect affiliation data for the later computation of social bonds, we recorded social behaviors during focal samples on an iPad 6. To this end, we created individual coding sheets using FileMaker Pro 15 (v.15.0.3.305). We recorded data on proximity, socially directed approaches, and social interactions (grooming and play). Proximity data were recorded during scan samples, every 5 min of the focal sampling. During each scan, we recorded the identity of the nearest neighbors (at arm's length distance) to the focal individual and the identity of the other present group members. The direction and outcome of socially directed approaches were recorded continuously, determining if a focal individual approached another group member or was approached by one; we recorded only positive (resulting in a social interaction or contact-sitting) or neutral (resulting in proximate sitting) approaches. In addition, we continuously recorded the duration and directionality (who initiated the social interaction) of social grooming and play between a focal individual and a partner.

To collect data for the later computation of rank differences, we recorded the outcome of agonistic interactions *ad libitum* (any time, in or outside of focal samples)*,* noting the IDs of involved individuals and assigned winner and loser, or recorded whether the interaction ended without a clear winner and loser, i.e. tie).

#### Design: definition and coding of entry and exit phases

To code the entry and exit phases of social play, grooming and mixed interactions (i.e., in which partners switched between grooming and/or play), we used the ELAN software package, v. 5.0 (Wittenburg et al., 2006). We analyzed 1,242 interactions that included 1,242 beginnings, and 1,240 endings. Some of these beginnings (*N* = 27) and endings (*N* = 45) were of insufficient visibility and could therefore not be further analyzed, leaving a final dataset of *N* = 1,215 visible interaction beginnings and *N* = 1,195 visible interaction endings (Table 1, results).

We defined an entry phase as the process by which partners recruit each other, via mutual gaze exchanges and intentional communicative signals (for intentionality criteria, see Byrne et al., 2017) to determine the type of the activity, negotiate its temporal and spatial properties and potentially establish joint commitment. Because to enter a joint action, both partners need to mutually agree to it, entries are always achieved through (at minimum) the exchange of mutual gaze (i.e., both partners simultaneously look at each other's face), but could also include activity-specific and species-specific, intentionally used initiation signals, such as gestures (see Byrne et al., 2017), vocalizations (Clay et al., 2015; see de Waal, 1988), and facial expressions (de Waal, 1988). For example, great apes use gestures (single or combined in sequences) to initiate play (Fröhlich et al., 2016a, 2016b), social grooming (Pika and Mitani, 2009) and joint travel between mothers and infants (Halina et al., 2013). If partners did not engage in mutual gaze exchanges and did not produce any other communicative signal before engaging in the activity itself, the entry phase was coded as absent. The duration of an entry phase was coded from the onset of the first mutual gaze was exchanged, until the onset of movements typical of the activity, i.e., first grooming movement with hands or mouth on partner's body for grooming, first body contact for contact play, and first running movement for pursuit of partner for chase play. Examples for entry phases can be found in the SI (Video S1, S2, S3, and S4).

We defined an exit phase as the process by which partners reach the mutual conviction that they are both ready to terminate the activity and, as a result, take leave of each other. They may express intentions to end a joint action via intentional communicative signals or other specific behaviors before walking away. Because we were unsure whether apes take into account their partner when disengaging from a joint action and whether they feel the need to reach a mutual agreement, we used a more inclusive definition for exit phases than entry phases (that were always “mutual”), and categorized exit types according to their degree of “sharedness” based on the type of gazes exchanged. We classified exits as “unilateral” if only one partner gazes at the other's face before separating, as “bilateral” if both partners gaze at each other's face but not simultaneously, as “mutual” if partners look at each other's faces simultaneously, or if one partner gazes at the other but the other uses tactile gestures, and as “signals-only” if individuals used no gaze but any other communicative signals (gestures, vocalizations, facial expressions). If partners did not engage in gaze exchanges or did not produce any other communicative signal before leaving their partner, the exit phase was coded as absent. The duration of an exit phase was coded from the moment all body movements typical of the activity ceased (e.g., movements of the hands on the body of the partner typical for grooming, and wrestling with, tickling or chasing each other for social play) until the moment that at least one partner gazed at the other before or while leaving (and at most after each partner gazed at each other once before or while separating), and if the interaction was not followed by a renewed interaction or attempt to re-engage partner within 2 min. Examples for exit phases can be found in the SI (Video S5, S6, S7, S8, S9, and S10).

We additionally recorded the IDs of the initiators of entry and exit phases and that of their interacting partner. Initiators were those individuals who approached and recruited a partner in the entry phase, and those who first initiated leaving movements or the first who stopped the activity-typical movements in exit phases.

We assessed inter-observer agreement for a combination of *both* the presence and duration of entry and exit phases using the EasyDIAg software package (Holle and Rein, 2015). The software allows to assess the agreement of raters in ELAN by detecting whether the raters agreed on the annotation value and duration. To reach agreement, the annotation values had to be identical and the duration had to overlap by at least 60%. The reliability agreement is thus based on a combined measure of annotation value and duration. The test revealed good agreement between RH and EG for bonobos (*N* = 38 interactions with entries and exits, Cohen's κ = 0.71) and moderate agreement between RH and EG for chimpanzees (*N* = 27 interactions with entries and exits Cohen's κ = 0.55). There was also good agreement between AP and EG for chimpanzees (*N* = 20 interactions with entries and exits, Cohen's κ = 0.71) and moderate agreement between LP and EG for chimpanzees (*N* = 33 interactions, Cohen's κ = 0.55). Our great apes' communication expert (EG) carefully double-checked and discussed with the second coders the cases where agreement was not met. Once agreement was met, those cases were corrected.

#### Social relations

As an inverse proxy for social distance, we computed the social affinity or social bond strength between partners, the Dyadic Composite Sociality Index (DSI) (Silk et al., 2013) using the socialindices package in R (https://github.com/gobbios/socialindices). The DSI is defined as Equation 1, where *d* comprises the numbers (or duration) of behaviors chosen to select the index, *f_ixy_* denotes the rate (or duration) of behavior *i* for the dyad *xy*, and *f_i_* constitutes the mean rate (or mean duration) of that behavior *i* considering all dyads of the sample (Silk et al., 2013).

(Equation1) $$DSI_{xy}=\frac{\sum_{i=1}^{d}\frac{f_{ixy}}{f_{i}}}{d}$$

DSI values range from 0→∞; larger values indicate stronger social bonds, and lower values indicate weaker social bonds. Our computation included the measures of grooming and play duration between two partners, their proximity rates (i.e., how frequently they were within arm's reach distance from one another) and their approach rates (i.e., focal having approached the partner or the reverse). Because the bonobos of San Diego Zoo were not always let out in a group on the same day, we additionally controlled for observation time and co-residency of dyads for this group.

As a proxy for power differences between social partners, we computed rank differences with Elo-ratings for individuals of each site using the Elo-Rating package in R (Neumann and Kulik, 2014). Elo-ratings are based on the outcomes of agonistic interactions between partners and depend on the sequence in which interaction take place over time. At the start of the rating procedure, each individual is assigned the same arbitrary value (e.g., 1,000), and the rating of the two interacting individuals will be updated according to the outcome of their conflict; winners gain points, losers lose points, with the amount depending on the expected probability that the higher-rated individual wins. We assessed wins and losses based on the following behaviors: aggressing an individual physically or by showing aggressive behaviors, taking away resources, displacing or chasing an opponent, or fleeing from the opponent, being displaced, showing submissive behaviors such as bared-teeth displays or whimpers, or giving away a resource. Outcomes were considered undecided if no clear winner or loser could be determined (e.g., both showing aggressive behaviors, chasing each other, hitting or slapping another, sharing the resource upon negotiation). Such undecided outcomes are incorporated as a disadvantage to higher-ranked individuals (a decrease of rating, but smaller than for a lost conflict). Since Elo-ratings closely reflect traditional ordinal dominance ranks, for simplicity we refer to Elo-ratings as ranks and Elo-rating differences as rank differences. To obtain rank differences, we subtracted the rank of the partner from the rank of the phase initiator: negative rank differences then indicated that the initiator of the phase was lower-ranked than the partner, and positive rank differences indicated that the initiator of the phase was higher-ranking to the partner.

#### Quantification and statistical analysis

To test our predictions, we fitted Bayesian generalized mixed models (*b*GLMMs) and Bayesian linear mixed effect models (*b*LMMs) using the Stan computational framework (http://mc-stan.org/), accessed through the brms package (Bürkner, 2017) in R v. 3.6.1 (R Core Team, 2013). Each model included four chains relying on Markov chain Monte Carlo estimation (MCMC), with 10,000 iterations per chain, of which we specified 2,000 iterations as warm-up to ensure sampling calibration. The model diagnostics revealed that the posterior distributions reflect the distribution of the original response values appropriately, R-hat statistics were <1.05, the number of effective samples was >100, and the MCMC chains had no divergent transitions (Table S2; Figure S2–S6). For all models, we used the default priors of the brms package (v. 2.9.0), which were weakly informative with a student's *t*-distribution of 3 degrees of freedom and a scale parameter of 10 (see Table S2). For inference, we calculated 95% credible intervals (CrI) from the posterior distributions and checked whether 0 was comprised in this interval. In all models, we controlled for age differences and sex differences by including age difference and whether the dyad was same-sex as covariates.

To analyze whether entry and exit phases are affected by social bond strength and rank difference, we first fitted *b*GLMMs for the presence of entry phases (fitting a Bernoulli distribution with binary dependent variable, model 1) and exit phases (binary outcome, model 3). Additionally, we fitted *b*LMMs for the duration of entry phases (fitting a lognormal distribution, model 2) and exit phases (model 4). For each of these models (see Table S2), we fitted fixed effects for social bond strength (DSI; as a measure of social distance), rank difference (as a measure of power difference), species, age difference in years, sex difference (same or different sex), interaction effects between social bond strength and species, and interaction effects between rank difference and species. Additionally, we fitted crossed random effects with initiator and partner ID. We z-transformed both social bond strength and rank difference for all individuals (both species).

To assess the possibility of subject variation with respect to the effects of DSI and rank difference, we fitted a second version of models 1–4 (Table S5; Figure S7–S10), using random slopes for initiators' and partners' social bond strength and rank differences, and compared those models to their first version (excluding the random slopes) using leave-one-out cross-validation (Vehtari et al., 2015) (Table S6). Since the differences in the expected log predictive density (ELPD) between slope and intercept models were much smaller than twice the estimated standard error, the random slope models were not expected to have a better predictive performance than the intercept only models. In addition, low sample sizes on subject level can lead to unreliable subject-level slope estimates (i.e., for entry and exit phases respectively, where 11 and 17 initiators had interactions with maximally four partners). We therefore only present our primary analysis without subject-level slopes.

To test whether the engagement in mutual exits (i.e., involves cases of mutual gaze, or unilateral gaze from one and production of tactile gesture by the other partner) depends on species and activity type, we fitted a *b*GLMM (model 5; Table S2), including as dependent variable the presence or absence of mutual exits (binary outcome), as fixed effects age difference in years, sex difference (i.e., same or different sex), species, and activity type (i.e., play, mixed, grooming). Additionally, we fitted crossed random intercepts for exit phase dyad ID. For the purpose of assessing the mutual responsiveness typical of joint commitment in humans, we were particularly interested in assessing the likelihood of mutual exits (i.e., mutual gazes or unilateral gaze and touch) versus non-mutual exits (including all other gaze classes) in apes.

Further details on the datasets used for these analysis can be found in the Data S1.

#### Additional information about datasets used for Bayesian mixed models 1–5

Due to the incapability of determining a clear phase initiator for mutually initiated exit phases, we could not integrate *N* = 66 observations of *mutually initiated* exit phases in any of our models. For the analysis of exit phase presence (model 3), we considered the total of *N* = 1,195 interaction endings, but excluded *N* = 66 data points of mutually terminated activities, as we were unable to determine a clear “terminator” and hence a direction of rank difference between partners. This yielded a final dataset of *N* = 1,129 exits in model 3. Next, we fitted *b*LMMs for the log duration (measured in seconds) of entry phases (model 2), and exit phases (model 4). For the analysis of entry phase duration (model 2), we considered only cases where entries were present; thus, from the total of *N* = 1,215 interaction beginnings, we excluded *N* = 294 data points where entries were absent (no duration measurable), yielding a final dataset of *N* = 921 entries in model 2. For the analysis of exit phase duration (model 4), we considered only cases where exits were present; thus, from the total of *N* = 1,195 interaction endings, we excluded *N* = 142 data points where exits were absent (no duration measurable), and further *N* = 51 remaining data points of mutually terminated activities, yielding a final dataset of *N* = 1,002 exits in model 4.

In the dataset used to test whether the engagement in mutual exits depends on species and activity type (model 5), we were unable to include exit types that; a) were cases of the pilot study for which no exit gaze categories had been coded (*N* = 28), b) where no gaze category could be coded due to the absence of an exit (*N* = 142), and c) where gaze categories could not be determined because one of the two partners was not sufficiently visible to assess whether or not mutual gaze existed (*N* = 42). This meant that out of a total dataset of *N* = 1,195 interaction endings (see Table 1 results), we could analyze mutual exits from a final dataset of *N* = 983 exits (Table S2).

## Supplemental information

Document S1. Figures S1–S10 and Tables S1–S6

Data S1. Full dataset supporting the article's findings

## Footnotes

Supplemental information can be found online at https://doi.org/10.1016/j.isci.2021.102872.
