# Supplementary material for: Assessing joint commitment as a process in great apes
Source: iScience. 2021 Aug 11;24(8):102872. doi: 10.1016/j.isci.2021.102872 (PMC8390869; doi:10.1016/j.isci.2021.102872)
Supplement: Document S1. Figures S1–S10 and Tables S1–S6 [file mmc1.pdf]

iScience, Volume *24*

# Supplemental information

## Assessing joint commitment as a process in great apes

**Raphaela Heesen, Adrian Bangerter, Klaus Zuberbühler, Katia Iglesias, Christof Neumann, Aude Pajot, Laura Perrenoud, Jean-Pascal Guéry, Federico Rossano, and Emilie Genty**

# Supplementary Information

**Figure S1. Variation of mutual exits across species and activity type (model 5).** Plots portray the predicted probability of mutual exits for the marginal effects of species (A) and activity type (B) for the Bayesian generalized linear mixed model 5 and show how the model fits the actual data (circles in grey). The upper and lower bars correspond to the upper and lower 95% credible intervals, respectively, and the squares represent the posterior means.  Each circle corresponds to the proportion of mutual exits present per interaction dyad, out of each dyad's total number of exits. Circle size corresponds to the total number of observed exits per dyad. Related to Figure 2 and Figure 4.

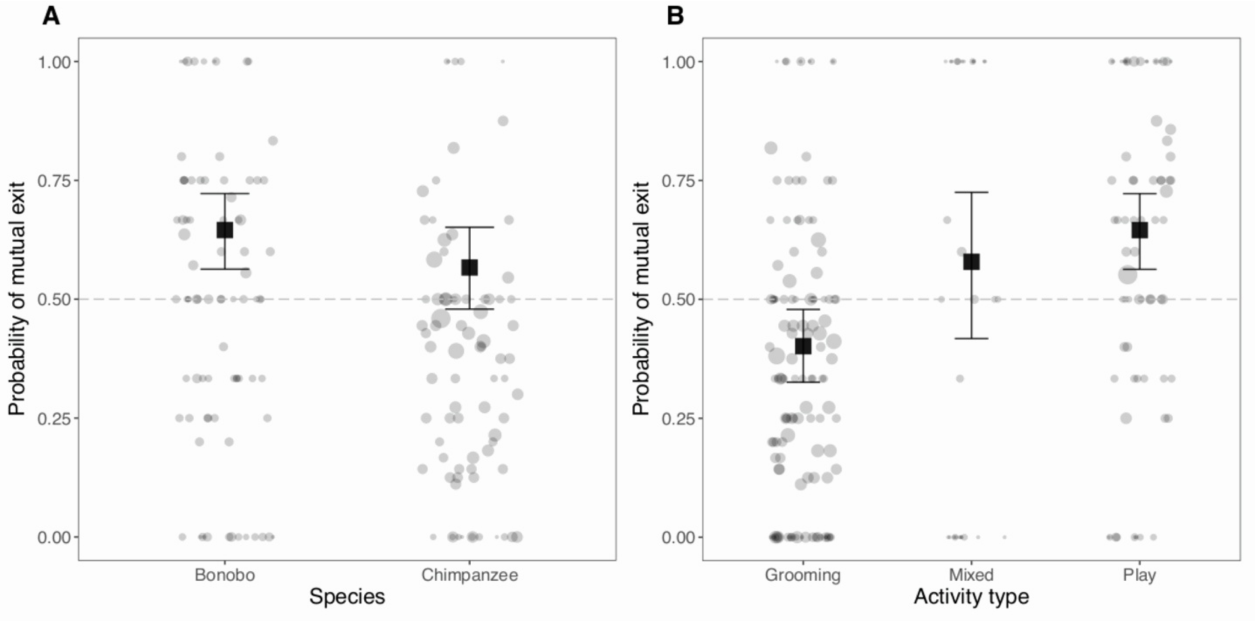

**Figure S2.** Compiled panels of posterior distributions of model 1 (entry phase presence) and MCMC trace-plots of selected Bayesian GLMMs and LMMs (with random intercepts). Related to Figure 1.

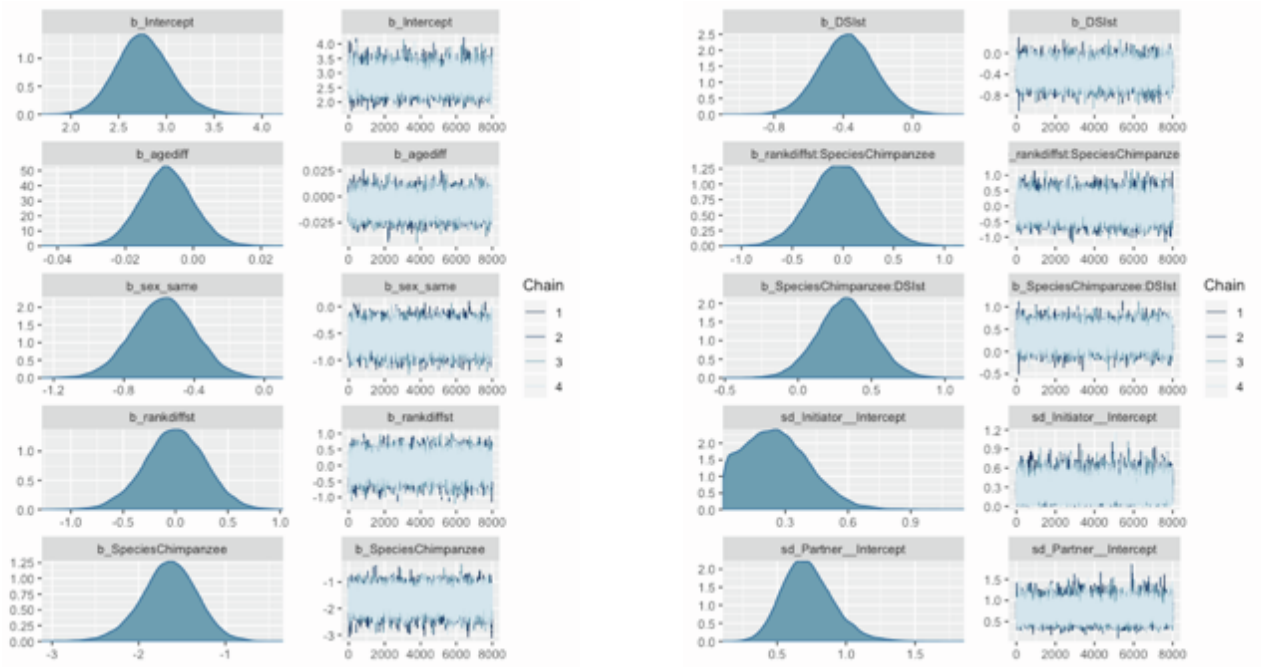

**Figure S3.** Compiled panels of posterior distributions of model 2 (entry phase duration) and MCMC trace-plots of selected Bayesian GLMMs and LMMs (with random intercepts). Related to Figure 3.

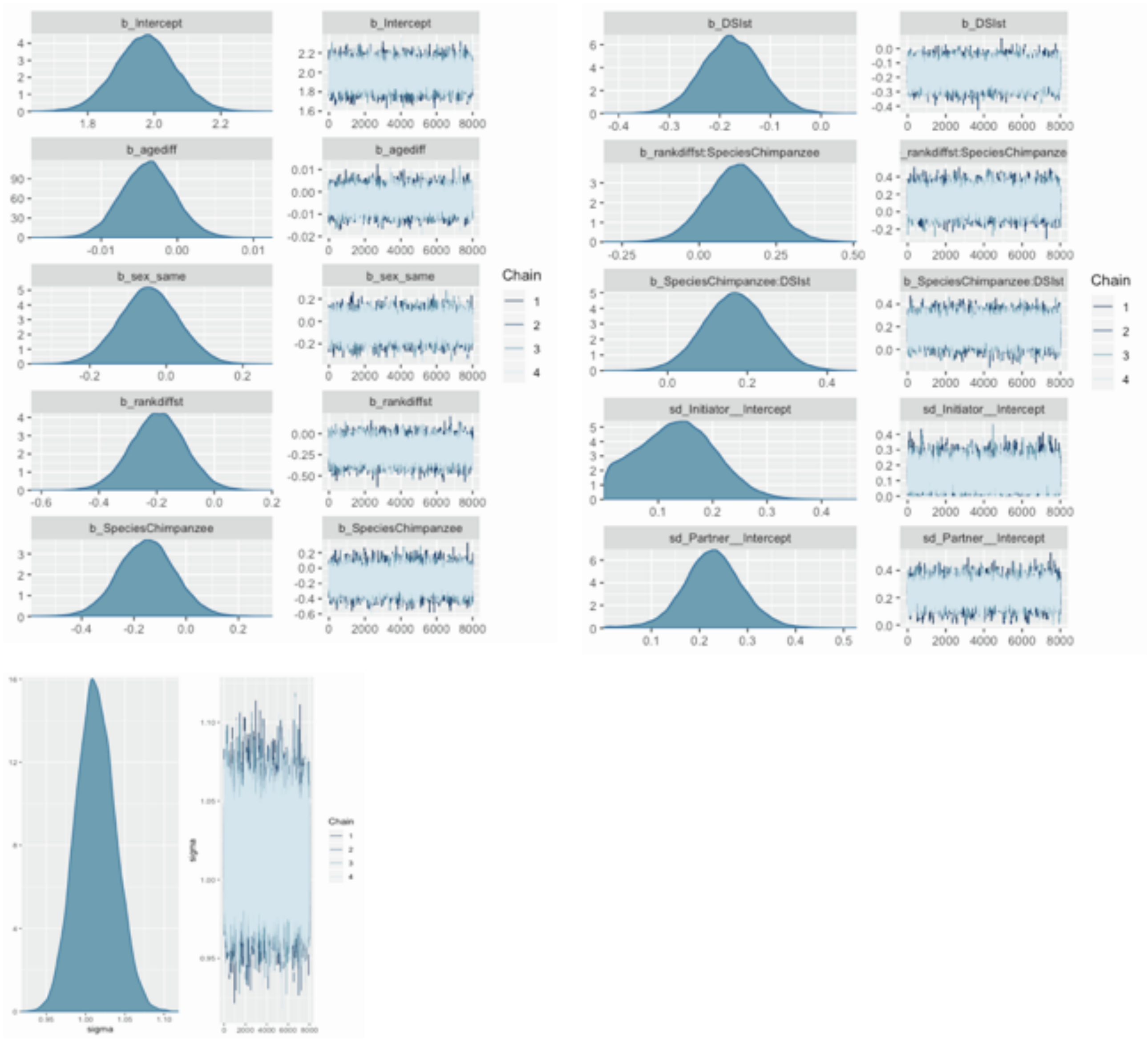

**Figure S4.** Compiled panels of posterior distributions of model 3 (exit phase presence) and MCMC trace-plots of selected Bayesian GLMMs and LMMs (with random intercepts). Related to Figure 2.

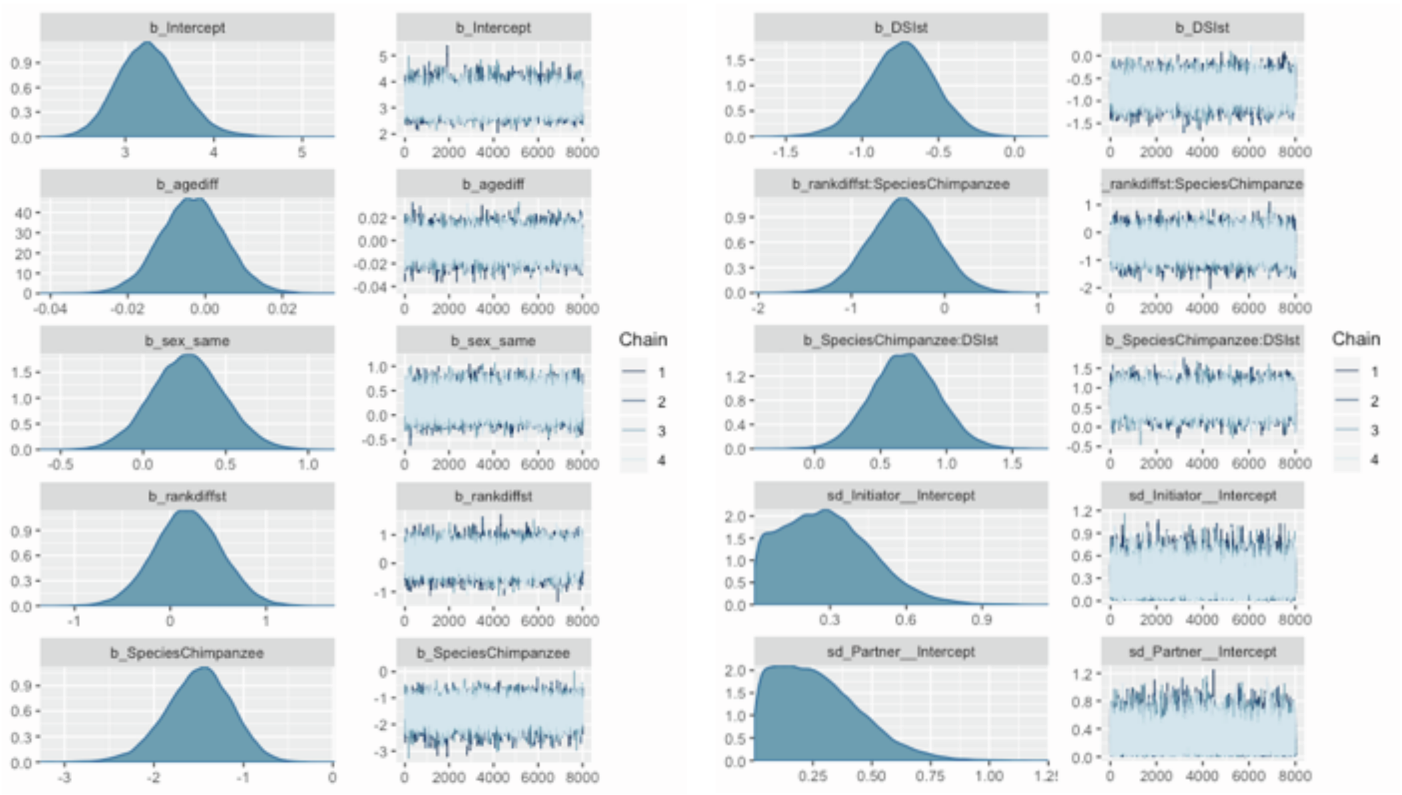

**Figure S5.** Compiled panels of posterior distributions of model 4 (exit phase duration) and MCMC trace-plots of selected Bayesian GLMMs and LMMs (with random intercepts). Related to Figure 4.

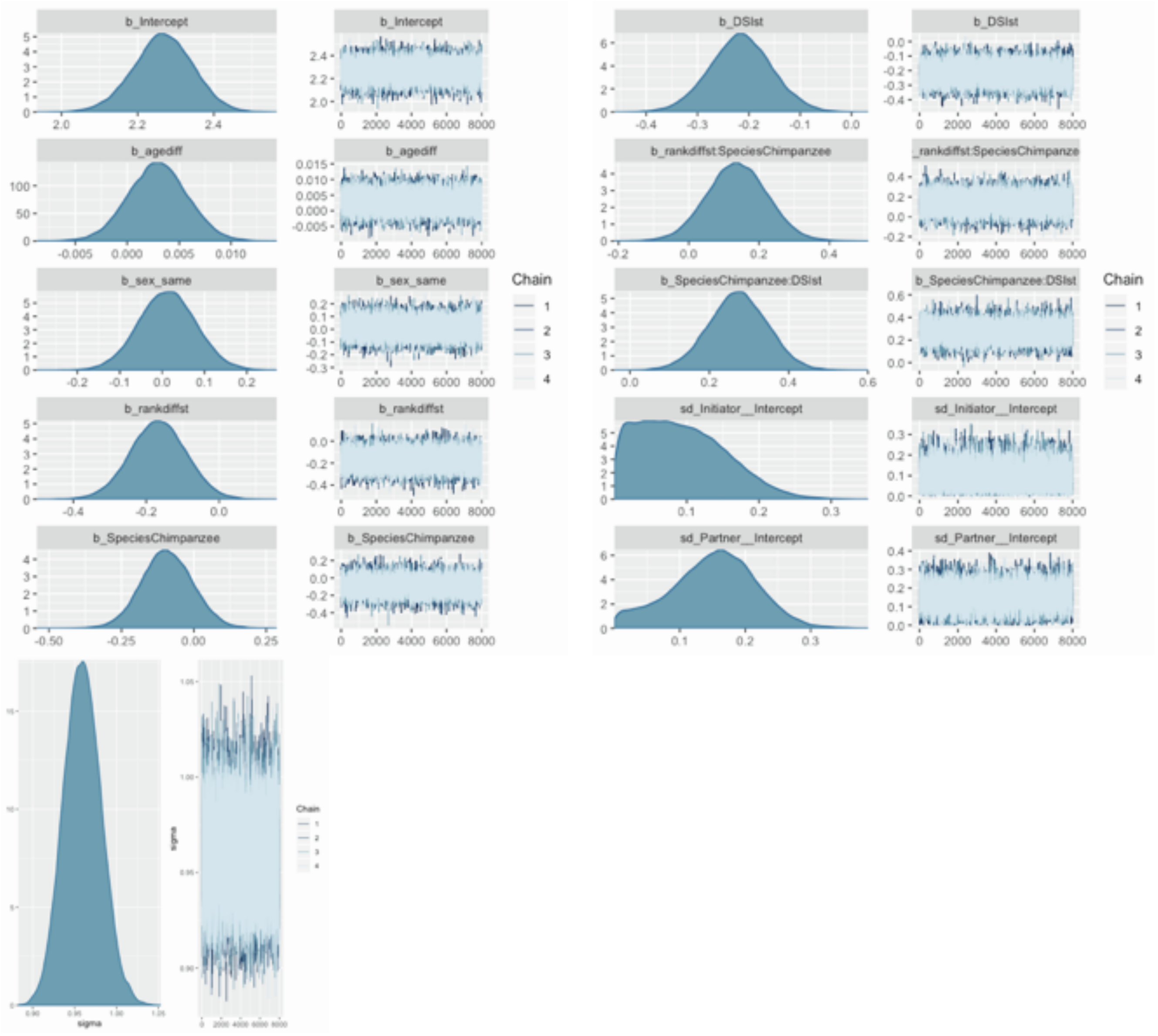

**Figure S6.** Compiled panels of posterior distributions of model 5 (engagement in mutual exit types) and MCMC trace-plots of selected Bayesian GLMMs and LMMs (with random intercepts). Related to Figures 2 and 4 and Figure S1.

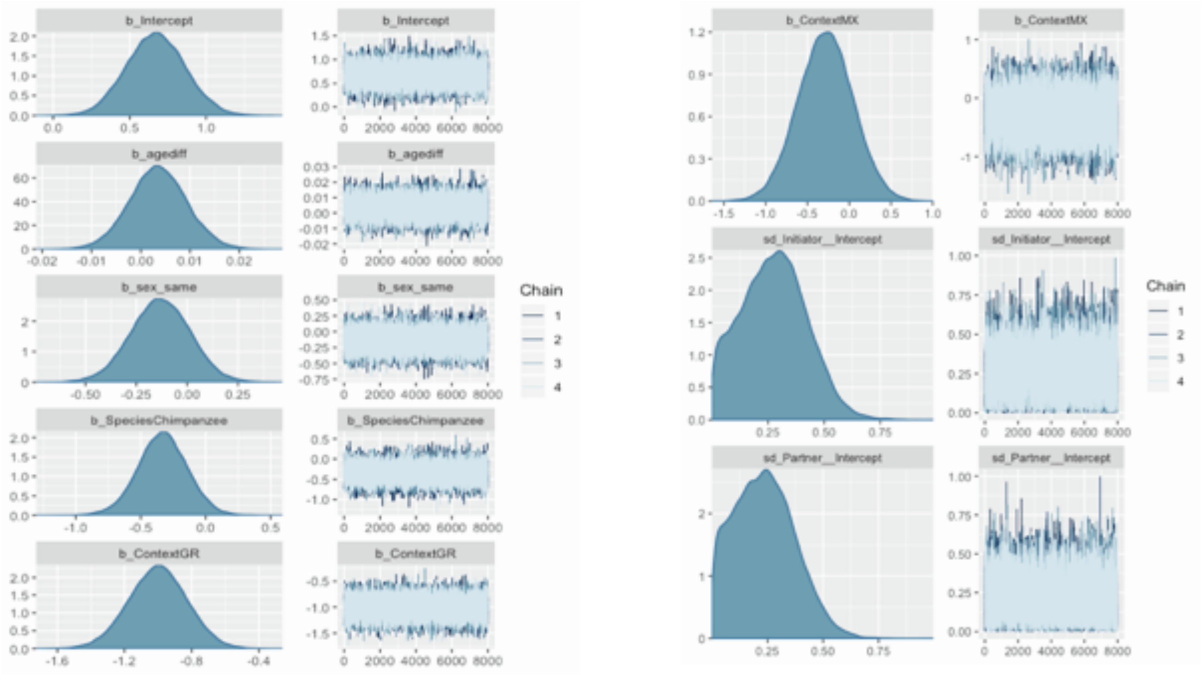

**Figure S7.** Compiled panels of posterior distributions of model 1 (entry phase presence) and MCMC trace-plots of *non-selected* Bayesian GLMMs and LMMs (with random slopes). Related to STAR methods and Table S5.

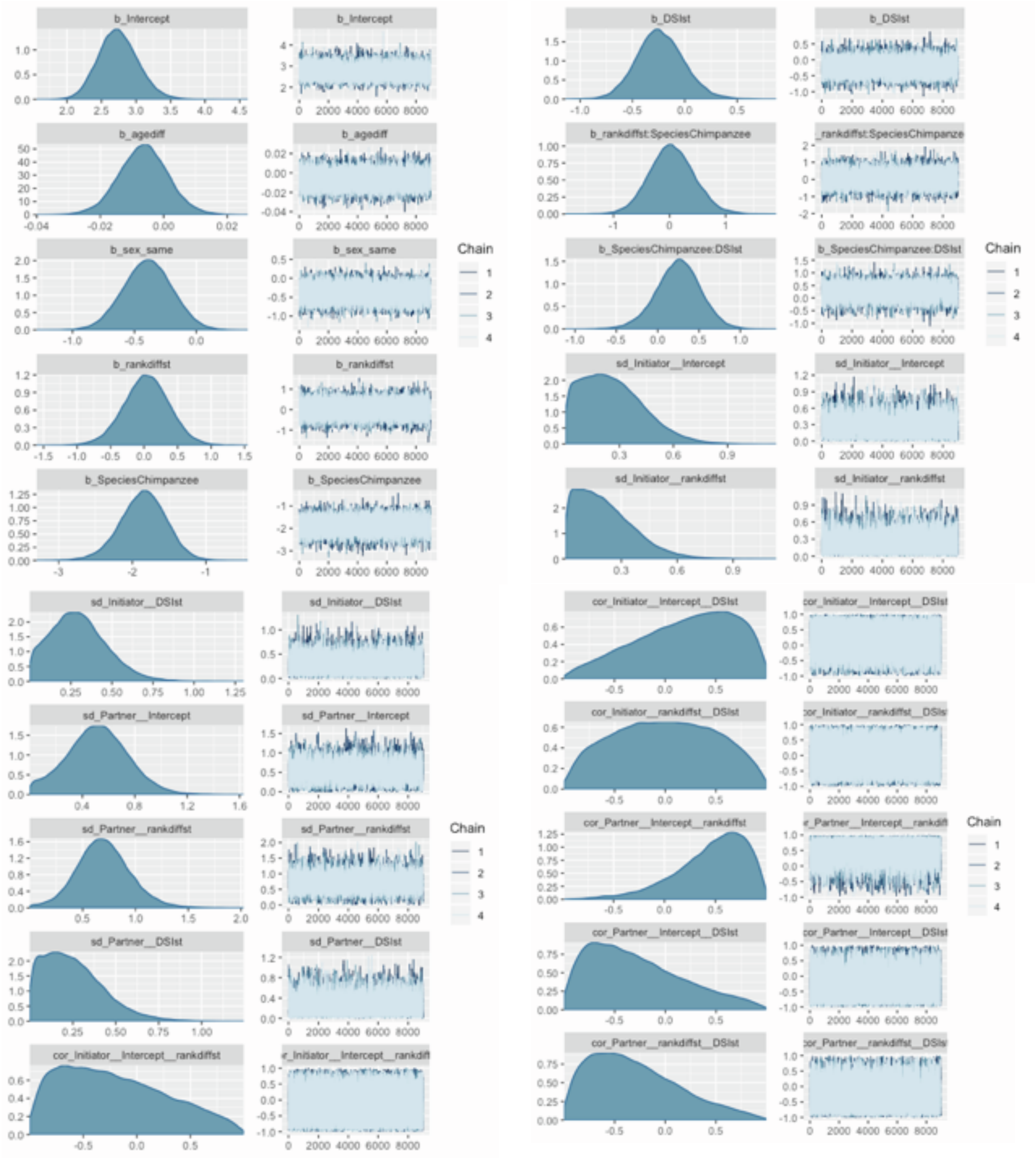

**Figure S8.** Compiled panels of posterior distributions of model 2 (entry phase duration) and MCMC trace-plots of *non-selected* Bayesian GLMMs and LMMs (with random slopes). Related to STAR methods and Table S5.

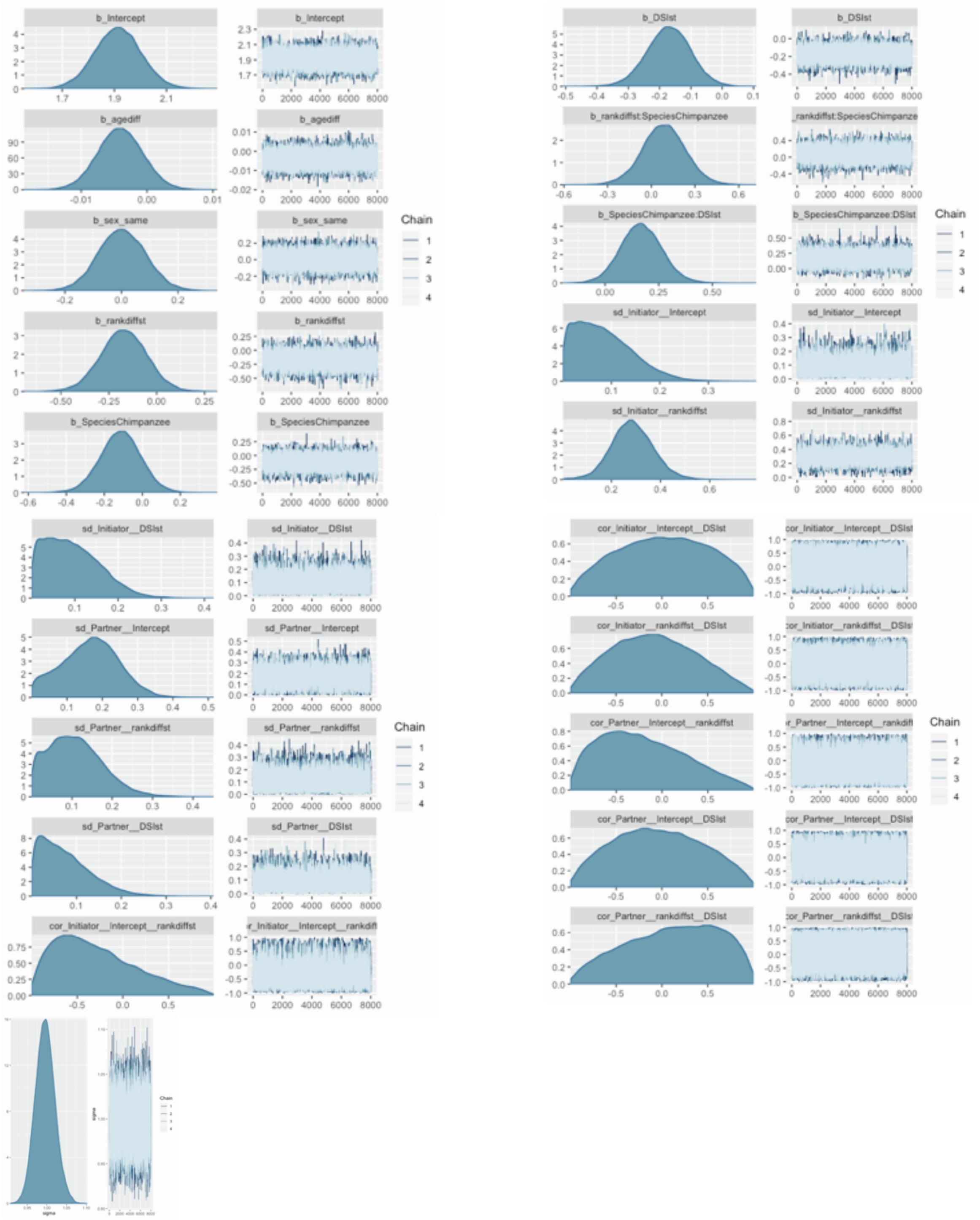

**Figure S9.** Compiled panels of posterior distributions of model 3 (exit phase presence) and MCMC trace-plots of *non-selected* Bayesian GLMMs and LMMs (with random slopes). Related to STAR methods and Table S5.

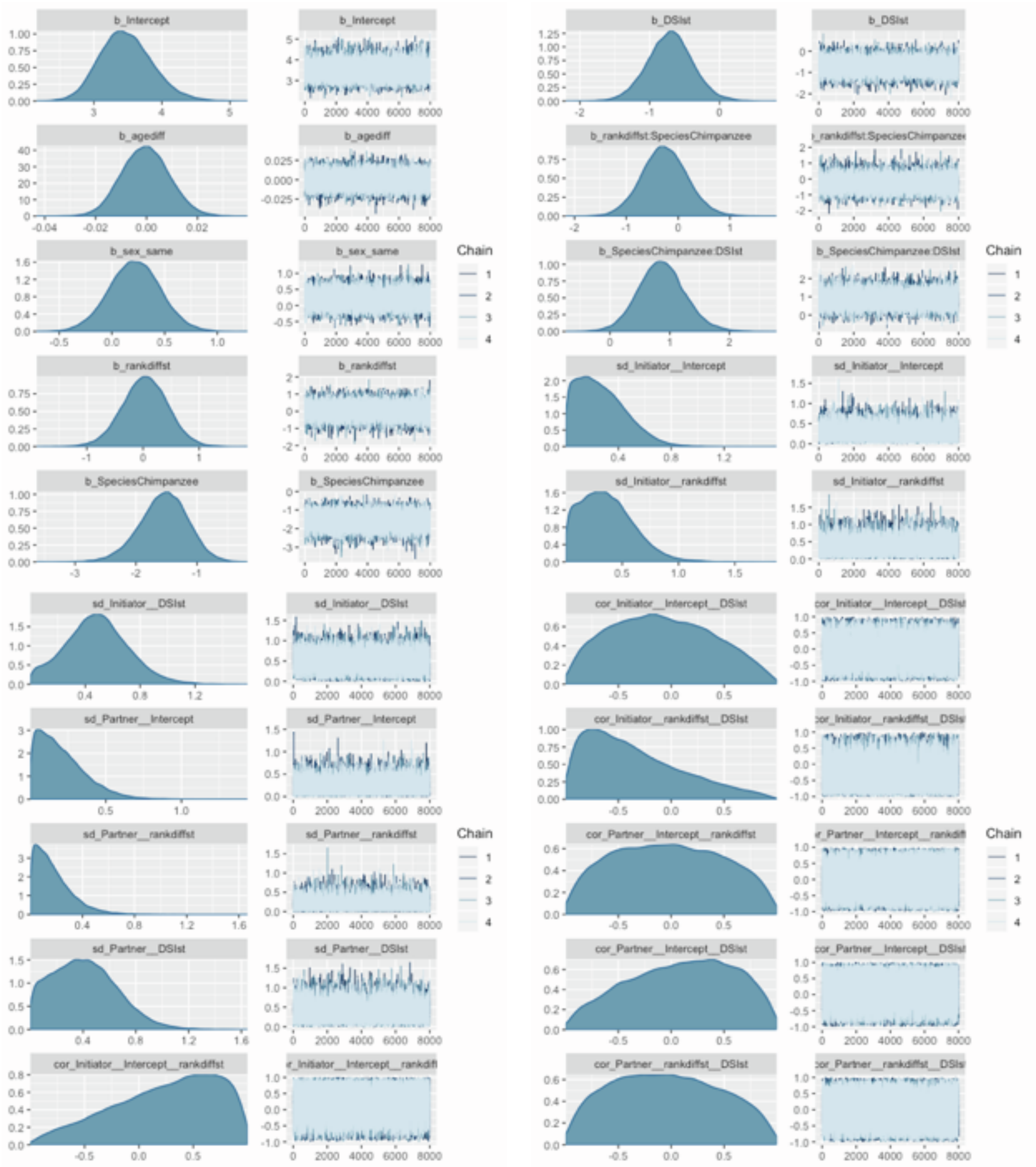

**Figure S10.** Compiled panels of posterior distributions of model 4 (exit phase duration) and MCMC trace-plots of *non-selected* Bayesian GLMMs and LMMs (with random slopes). Related to STAR methods and Table S5.

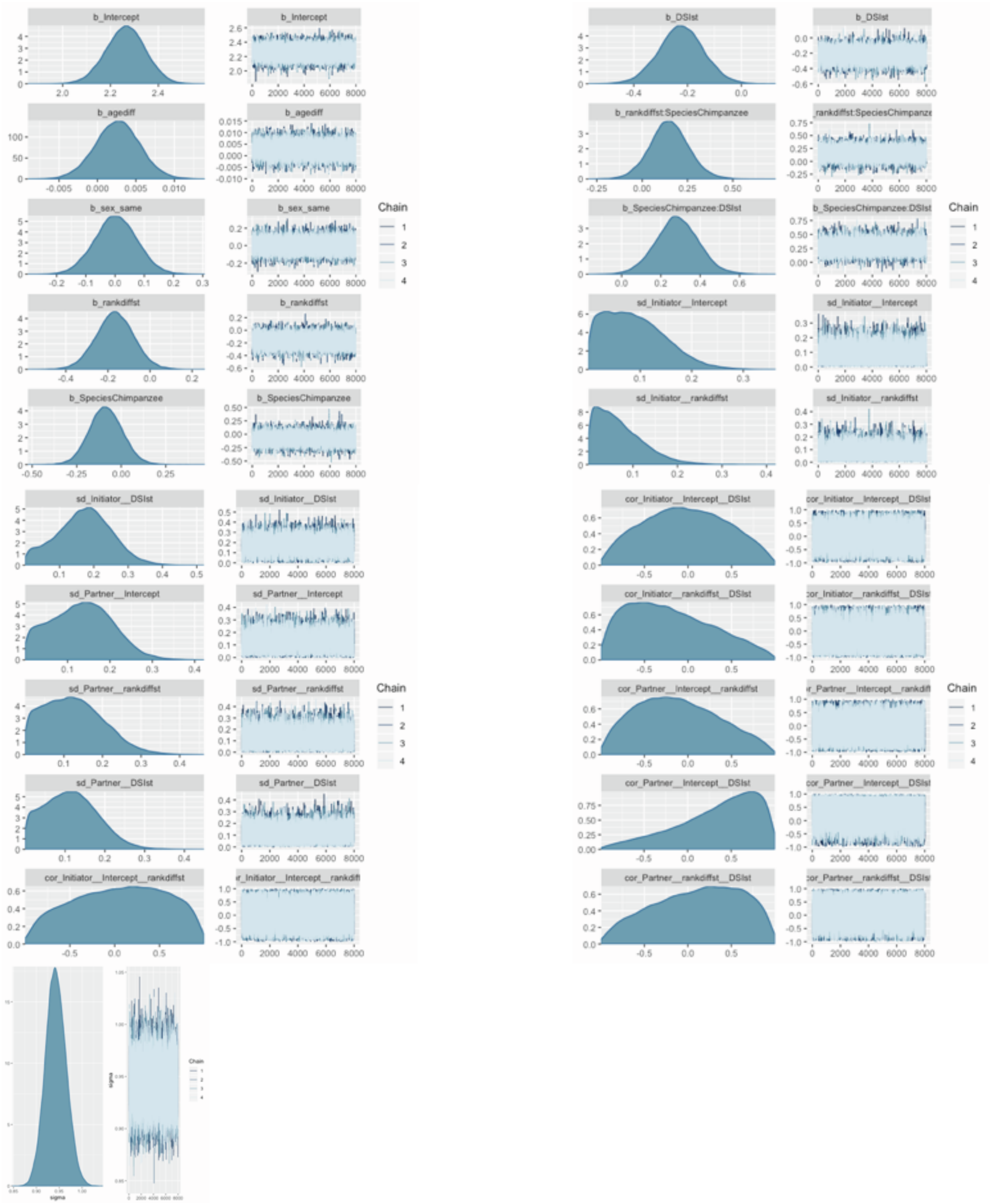

**Table S1.** Descriptive statistics of entry and exit duration in bonobos and chimpanzees. Related to Table 1.

| Phase | Species | Mean (s) | SD (s) | Median (s) | IQR[1] |
|---|---|---|---|---|---|
| Entry | Bonobo | 12.7 | 20.2 | 5.9 | 3.4; 14.4 |
| Entry | Chimpanzee | 11.5 | 21.0 | 6.4 | 3.0; 12.0 |
| Exit | Bonobo | 16.8 | 22.3 | 9.2 | 5.0; 18.5 |
| Exit | Chimpanzee | 13.6 | 14.6 | 9.0 | 4.8; 16.9 |

*Note*. [1]IQR= Interquartile range with lower (1$^{st}$) and upper (3$^{rd}$) quartile

**Table S2.** Summary of results for selected Bayesian GLMMs and LMMs (without random slopes). Related to Figures 1-4 and Figure S1.

| Model 1: Entry phase presence (*N*=1215) | | | | | | |
|---|---|---|---|---|---|---|
| *Fixed effects* | ***b*** | ***SE*** | **95% CrI** | ***Eff. N*** | $\hat{R}$ | ***Default Prior***[A] |
| Intercept | 2.77 | 0.29 | [2.22;3.37] | 19147 | 1.00 | Student *t* (3,0,10) |
| Species | | | | | | |
| Bonobo | *Reference* | | | | | |
| Chimpanzee | -1.65 | 0.32 | [-2.32;-1.04] | 16661 | 1.00 | Student *t* (3,0,10) |
| Age difference in years | -0.01 | 0.01 | [-0.02;0.01] | 22506 | 1.00 | Student *t* (3,0,10) |
| Sex | | | | | | |
| Different | *Reference* | | | | | |
| Same | -0.57 | 0.18 | [-0.92;-0.23] | 32492 | 1.00 | Student *t* (3,0,10) |
| DSI | -0.38 | 0.16 | [-0.69;-0.06] | 21819 | 1.00 | Student *t* (3,0,10) |
| Rank difference | -0.01 | 0.29 | [-0.59;0.55] | 16592 | 1.00 | Student *t* (3,0,10) |
| DSI * Species | 0.33 | 0.19 | [-0.05;0.70] | 20838 | 1.00 | Student *t* (3,0,10) |
| Rank difference * Species | -0.02 | 0.30 | [-0.61;0.57] | 20216 | 1.00 | Student *t* (3,0,10) |
| *Random effects* | | | | | | |
| Initiator | 0.26 | 0.15 | [0.01;0.59] | 6490 | 1.00 | Student *t* (3,0,10) |
| Partner | 0.72 | 0.18 | [0.40;1.12] | 7973 | 1.00 | Student *t* (3,0,10) |

| Model 2: Entry phase duration (*N* =921) | | | | | | |
|---|---|---|---|---|---|---|
| *Fixed effects* | ***b*** | ***SE*** | **95% CrI** | ***Eff. N*** | $\hat{R}$ | ***Default Prior***[A] |
| Intercept | 1.97 | 0.09 | [1.80;2.15] | 32827 | 1.00 | Student *t* (3,2,10) |
| Species | | | | | | |
| Bonobo | *Reference* | | | | | |
| Chimpanzee | -0.15 | 0.11 | [-0.36;0.06] | 27753 | 1.00 | Student *t* (3,2,10) |
| Age difference in years | -0.00 | 0.00 | [-0.01;0.00] | 30663 | 1.00 | Student *t* (3,2,10) |
| Sex | | | | | | |
| Different | *Reference* | | | | | |
| Same | -0.04 | 0.08 | [-0.20;0.11] | 47612 | 1.00 | Student *t* (3,2,10) |
| DSI | -0.18 | 0.06 | [-0.29;-0.06] | 33385 | 1.00 | Student *t* (3,2,10) |
| Rank difference | -0.19 | 0.09 | [-0.38;-0.01] | 16671 | 1.00 | Student *t* (3,2,10) |
| DSI * Species | 0.17 | 0.08 | [0.01;0.33] | 32688 | 1.00 | Student *t* (3,2,10) |
| Rank difference * Species | 0.13 | 0.10 | [-0.07;0.33] | 20834 | 1.00 | Student *t* (3,2,10) |
| *Random effects* | | | | | | |
| Initiator | 0.13 | 0.07 | [0.01;0.27] | 7014 | 1.00 | Student *t* (3,0,10) |
| Partner | 0.23 | 0.06 | [0.10;0.35] | 8137 | 1.00 | Student *t* (3,0,10) |

| Model 3: Exit phase presence (*N* =1129) | | | | | | |
|---|---|---|---|---|---|---|
| *Fixed effects* | ***b*** | ***SE*** | **95% CrI** | ***Eff. N*** | $\hat{R}$ | ***Default Prior***[A] |
| Intercept | 3.28 | 0.36 | [2.63;4.03] | 19494 | 1.00 | Student *t* (3,0,10) |
| Species | | | | | | |
| Bonobo | *Reference* | | | | | |

| | | | | | | |
|---|---|---|---|---|---|---|
| Chimpanzee | -1.50 | 0.37 | [-2.26;-0.83] | 21585 | 1.00 | Student *t* (3,0,10) |
| Age difference in years | -0.00 | 0.01 | [-0.02;0.01] | 28826 | 1.00 | Student *t* (3,0,10) |
| Sex | | | | | | |
| Different | *Reference* | | | | | |
| Same | 0.27 | 0.22 | [-0.15;0.70] | 34391 | 1.00 | Student *t* (3,0,10) |
| DSI | -0.74 | 0.22 | [-1.18;-0.32] | 18007 | 1.00 | Student *t* (3,0,10) |
| Rank difference | 0.18 | 0.35 | [-0.49;0.86] | 20094 | 1.00 | Student *t* (3,0,10) |
| DSI * Species | 0.69 | 0.25 | [0.20;1.19] | 18864 | 1.00 | Student *t* (3,0,10) |
| Rank difference * Species | -0.44 | 0.36 | [-1.15;0.28] | 19765 | 1.00 | Student *t* (3,0,10) |
| | | | | | | |
| *Random effects* | | | | | | |
| Initiator | 0.29 | 0.17 | [0.01;0.66] | 7585 | 1.00 | Student *t* (3,0,10) |
| Partner | 0.27 | 0.18 | [0.01;0.69] | 7313 | 1.00 | Student *t* (3,0,10) |

| Model 4: Exit phase duration (*N* =1002) | | | | | | |
|---|---|---|---|---|---|---|
| *Fixed effects* | ***b*** | ***SE*** | **95% CrI** | ***Eff. N*** | $\hat{R}$ | ***Default Prior***[A] |
| Intercept | 2.27 | 0.08 | [2.11;2.42] | 29823 | 1.00 | Student *t* (3,2,10) |
| Species | | | | | | |
| Bonobo | *Reference* | | | | | |
| Chimpanzee | -0.09 | 0.09 | [-0.26;0.08] | 23105 | 1.00 | Student *t* (3,2,10) |
| Age difference in years | 0.00 | 0.00 | [-0.00;0.01] | 21687 | 1.00 | Student *t* (3,2,10) |
| Sex | | | | | | |
| Different | *Reference* | | | | | |
| Same | 0.01 | 0.07 | [-0.12;0.14] | 40400 | 1.00 | Student *t* (3,2,10) |
| DSI | -0.22 | 0.06 | [-0.34;-0.10] | 22748 | 1.00 | Student *t* (3,2,10) |
| Rank difference | -0.17 | 0.08 | [-0.32;-0.02] | 19435 | 1.00 | Student *t* (3,2,10) |
| DSI * Species | 0.27 | 0.07 | [0.13;0.42] | 22660 | 1.00 | Student *t* (3,2,10) |
| Rank difference * Species | 0.14 | 0.09 | [-0.03;0.31] | 19955 | 1.00 | Student *t* (3,2,10) |
| | | | | | | |
| *Random effects* | | | | | | |
| Initiator | 0.09 | 0.06 | [0.00;0.22] | 5608 | 1.00 | Student *t* (3,0,10) |
| Partner | 0.15 | 0.06 | [0.02;0.27] | 5020 | 1.00 | Student *t* (3,0,10) |

| Model 5: Use of mutual exit types (*N*=983) | | | | | | |
|---|---|---|---|---|---|---|
| *Fixed effects* | ***b*** | ***SE*** | **95% CrI** | ***Eff. N*** | $\hat{R}$ | ***Default Prior***[A] |
| Intercept | 0.67 | 0.19 | [0.30;1.05] | 44282 | 1.00 | Student *t* (3,0,10) |
| Species | | | | | | |
| Bonobo | *Reference* | | | | | |
| Chimpanzee | -0.33 | 0.19 | [-0.71;0.04] | 36127 | 1.00 | Student *t* (3,0,10) |
| Age difference in years | 0.00 | 0.01 | [-0.01;0.02] | 24425 | 1.00 | Student *t* (3,0,10) |
| Sex | | | | | | |
| Different | *Reference* | | | | | |
| Same | -0.13 | 0.15 | [-0.42;0.15] | 49520 | 1.00 | Student *t* (3,0,10) |
| Context | | | | | | |
| Play | *Reference* | | | | | |
| Grooming | -1.00 | 0.17 | [-1.35;-0.67] | 46006 | 1.00 | Student *t* (3,0,10) |
| Mixed | -0.28 | 0.33 | [-0.93;0.36] | 57367 | 1.00 | Student *t* (3,0,10) |

| | | | | | | |
|---|---|---|---|---|---|---|
| *Random effects* | | | | | | |
| Dyad | 0.24 | 0.14 | [0.01;0.52] | 7613 | 1.00 | Student *t* (3,0,10) |

*Note.* All datasets used for the mixed models excluded *N*=118 observations of exit phases that were identified as “mutually intitiated” phases, due to the problem of determining an ID of an initiator and partner for random effects.

[A]Bürkner, P.C. (2017). Brms: An R Package for Bayesian Multilevel Models Using Stan. *Journal of Statistical Software*, 80(1), 1-28. https://doi.org/10.18637/jss.v080.i01.

**Abbreviations**: b= Estimated mean of the posterior distribution; SD= Standard deviation of the posterior distribution; Crl= Two-sided 95% Credible intervals based on quantiles; $\hat{R}$=R hat value, provides information about the convergence of the MCMC algorithm - if larger than 1.1, chains have not converged and model is not accurate; Eff. N= number of effective sample size (i.e., number of independent samples from the posterior distribution, which would be expected to give the same standard error of posterior mean as obtained from dependent samples returned by MCMC algorithms).

**Table S3.** Occurrences of mutual exits across species and activity types. Related to Table 1 and Figure S1.

| | Bonobos (*N*=324 exits) | | | | Chimpanzees (*N*=659 exits) | | | | Total |
|---|---|---|---|---|---|---|---|---|---|
| | Play | Grooming | Mixed | Subtotal | Play | Grooming | Mixed | Subtotal | |
| Exits* | 134 | 176 | 14 | 324 | 134 | 490 | 35 | 659 | 983 |
| Of these, mutual exits | 80 | 74 | 11 | 165 | 82 | 158 | 15 | 255 | 420 |

*corresponding to exits used for mutual exit analysis, see methods.

**Table S4**. Information on site, species, focal individuals and hours of observation. Related to STAR methods.

| Site | Species | Focal ID | Birthdate | Sex | Hours observation |
|---|---|---|---|---|---|
| **San Diego Zoo, USA** | *Bonobo* | Belle | 2013-12-21 | F | *30* |
| | | Erin | 1991-12-23 | M | *30* |
| | | Kalli | 2005-03-15 | F | *30* |
| | | Lisa | 1991-06-14 | F | *30* |
| | | Loretta | 1974-01-22 | F | *30* |
| | | Maddie | 2009-03-24 | F | *30* |
| | | Makasi | 2004-04-22 | M | *30* |
| | | Mali | 2007-09-04 | F | *30* |
| | | Vic | 2001-06-27 | M | *30* |
| | | ***N* = 9** | | | ***270*** |
| **La Vallée des Singes, France** | *Bonobo* | Daniela | 1968-06-17 | F | *20.75* |
| | | David | 2001-07-27 | M | *20.25* |
| | | Diwani | 1996-08-11 | M | *20.25* |
| | | Kelele | 2004-07-22 | M | *22* |
| | | Khalessi | 2012-12-12 | F | *20.25* |
| | | Khaya | 2001-10-19 | F | *20.5* |
| | | Lingala | 2003-07-17 | F | *21* |
| | | Lokoro | 2015-05-22 | M | *21.25* |
| | | Loto | 2009-09-02 | M | *20.5* |
| | | Lucy | 2003-12-01 | F | *20.25* |
| | | Moko | 2012-08-04 | M | *20* |
| | | Swahili | 2014-09-21 | F | *20.75* |
| | | Ukela | 1985-12-19 | F | *20.25* |
| | | Ulindi | 1993-10-10 | F | *21* |
| | | Yahimba | 2009-08-07 | F | *21* |
| | | Yuli | 2014-07-14 | F | 20.25 |
| | | ***N* = 16** | | | **330.25** |
| | *Chimpanzee* | Cauna | 2007-05-05 | F | *37.1* |
| | | Conan | 1996-03-20 | M | *37.4* |
| | | Jorg | 1995-01-01 | M | *37.1* |
| | | Lila | 2009-06-20 | F | *37.4* |
| | | Panya | 2008-07-14 | F | *36.1* |
| | | Roy | 1997-09-27 | M | *37.2* |
| | | Wonder | 1997-03-19 | M | *36.8* |
| | | ***N* = 7** | | | ***259.1*** |
| **Basel Zoo, Switzerland** | *Chimpanzee* | Benga | 1979-09-17 | F | *30* |
| | | Colebe | 2005-07-12 | M | *30* |
| | | Fahamu | 2008-08-19 | M | *30* |
| | | Fifi | 1993-05-12 | F | *30* |
| | | Garissa | 2009-04-06 | F | *30* |
| | | Jacky | 1967-06-30 | F | *30* |
| | | Kitoko | 1993-05-20 | F | *30* |

| | | | | | |
|---|---|---|---|---|---|
| | | Kume | 2003-10-04 | M | *30* |
| | | Xindra | 1975-10-23 | F | *30* |
| | | ***N* = 9** | | | ***270*** |
| **La Réserve Africaine de Sigean, France** | *Chimpanzee* | Ann | 2002-07-21 | F | *18.9* |
| | | Goldie | 1989-08-29 | F | *19* |
| | | Inongo | 2009-06-19 | M | *18.8* |
| | | Jessica | 1973 | F | *18.8* |
| | | Macourie | 1999-12-12 | F | *18.6* |
| | | Madingo | 2013-09-02 | M | *17.8* |
| | | Pablo | 1999-11-12 | M | *19.3* |
| | | Penny | 1989 | F | *19.3* |
| | | Poppy | 2013-04-13 | F | *18.7* |
| | | ***N=9*** | | | ***169.2*** |
| **Grand total** | | ***N= 50*** | | | 1298.55 |

**Table S5**. Summary of results for non-selected Bayesian GLMMs and LMMs including random slopes. Related to STAR methods.

| Model 1 (RQ3): Entry phase presence (*N* =1215) | | | | | | |
|---|---|---|---|---|---|---|
| *Fixed effects* | ***b*** | ***SE*** | **95% CrI** | ***Eff. N*** | $\hat{R}$ | ***Default Prior***[A] |
| Intercept | 2.74 | 0.29 | [2.21;3.34] | 31901 | 1.00 | Student *t* (3,0,10) |
| Species | | | | | | |
| Bonobo | *Reference* | | | | | |
| Chimpanzee | -1.85 | 0.31 | [-2.50;-1.27] | 29561 | 1.00 | lkj_corr_cholesky(1) |
| Age difference in years | -0.01 | 0.01 | [-0.02;0.01] | 35215 | 1.00 | lkj_corr_cholesky(1) |
| Sex | | | | | | |
| Different | *Reference* | | | | | |
| Same | -0.39 | 0.20 | [-0.78;-0.01] | 41689 | 1.00 | lkj_corr_cholesky(1) |
| DSI | -0.23 | 0.23 | [-0.66;0.22] | 21188 | 1.00 | lkj_corr_cholesky(1) |
| Rank difference | 0.04 | 0.34 | [-0.65;0.70] | 25335 | 1.00 | lkj_corr_cholesky(1) |
| DSI * Species | 0.25 | 0.27 | [-0.31;0.77] | 23474 | 1.00 | lkj_corr_cholesky(1) |
| Rank difference * Species | 0.04 | 0.40 | [-0.72;0.85] | 24064 | 1.00 | lkj_corr_cholesky(1) |
| | | | | | | |
| *Random effects* | | | | | | |
| Initiator | | | | | | |
| sd(intercept) | 0.26 | 0.17 | [0.01;0.65] | 7308 | 1.00 | Student *t* (3,0,10) |
| sd(rank difference) | 0.21 | 0.16 | [0.01;0.58] | 13244 | 1.00 | Student *t* (3,0,10) |
| sd(DSI) | 0.30 | 0.17 | [0.02;0.67] | 11603 | 1.00 | Student *t* (3,0,10) |
| cor(intercept, rank difference) | -0.20 | 0.49 | [-0.93;0.80] | 20565 | 1.00 | lkj_corr_cholesky(1) |
| cor(intercept, DSI) | 0.20 | 0.47 | [-0.78;0.92] | 14353 | 1.00 | lkj_corr_cholesky(1) |
| cor(rank difference, DSI) | -0.00 | 0.49 | [-0.87;0.87] | 16923 | 1.00 | lkj_corr_cholesky(1) |
| Partner | | | | | | |
| sd(intercept) | 0.52 | 0.23 | [0.07;0.98] | 5372 | 1.00 | Student *t* (3,0,10) |
| sd(rank difference) | 0.70 | 0.25 | [0.21;1.22] | 9339 | 1.00 | Student *t* (3,0,10) |
| sd(DSI) | 0.25 | 0.17 | [0.01;0.66] | 10171 | 1.00 | Student *t* (3,0,10) |
| cor(intercept, rank difference) | 0.46 | 0.35 | [-0.39;0.94] | 6201 | 1.00 | lkj_corr_cholesky(1) |
| cor(intercept, DSI) | -0.28 | 0.46 | [-0.93;0.74] | 16971 | 1.00 | lkj_corr_cholesky(1) |
| cor(rank difference, DSI) | -0.30 | 0.44 | [-0.93;0.69] | 19997 | 1.00 | lkj_corr_cholesky(1) |

| Model 2 (RQ3): Entry phase duration (*N* =921) | | | | | | |
|---|---|---|---|---|---|---|
| *Fixed effects* | ***b*** | ***SE*** | **95% CrI** | ***Eff. N*** | $\hat{R}$ | ***Default Prior***[A] |
| Intercept | 1.91 | 0.09 | [1.74;2.09] | 37544 | 1.00 | Student *t* (3,2,10) |
| Species | | | | | | |
| Bonobo | *Reference* | | | | | |
| Chimpanzee | -0.12 | 0.11 | [-0.33;0.09] | 35279 | 1.00 | lkj_corr_cholesky(1) |
| Age difference in years | -0.00 | 0.00 | [-0.01;0.00] | 3262 | 1.00 | lkj_corr_cholesky(1) |
| Sex | | | | | | |
| Different | *Reference* | | | | | |
| Same | 0.00 | 0.08 | [-0.16;0.17] | 20113 | 1.00 | lkj_corr_cholesky(1) |
| DSI | -0.17 | 0.07 | [-0.31;-0.03] | 30992 | 1.00 | lkj_corr_cholesky(1) |

| | | | | | | |
|---|---|---|---|---|---|---|
| Rank difference | -0.17 | 0.12 | [-0.41;0.06] | 22096 | 1.00 | lkj_corr_cholesky(1) |
| DSI * Species | 0.17 | 0.10 | [-0.02;0.36] | 30382 | 1.00 | lkj_corr_cholesky(1) |
| Rank difference * Species | 0.09 | 0.15 | [-0.21;0.38] | 24113 | 1.00 | lkj_corr_cholesky(1) |
| | | | | | | |
| *Random effects* | | | | | | |
| Initiator | | | | | | |
| sd(intercept) | 0.08 | 0.06 | [0.00;0.22] | 9990 | 1.00 | Student *t* (3,0,10) |
| sd(rank difference) | 0.28 | 0.09 | [0.12;0.46] | 8995 | 1.00 | Student *t* (3,0,10) |
| sd(DSI) | 0.09 | 0.06 | [0.00;0.23] | 10946 | 1.00 | Student *t* (3,0,10) |
| cor(intercept, rank difference) | -0.29 | 0.44 | [-0.93;0.72] | 4160 | 1.00 | lkj_corr_cholesky(1) |
| cor(intercept, DSI) | 0.03 | 0.49 | [-0.86;0.88] | 19216 | 1.00 | lkj_corr_cholesky(1) |
| cor(rank difference, DSI) | -0.08 | 0.45 | [-0.87;0.79] | 26116 | 1.00 | lkj_corr_cholesky(1) |
| Partner | | | | | | |
| sd(intercept) | 0.17 | 0.08 | [0.01;0.32] | 6950 | 1.00 | Student *t* (3,0,10) |
| sd(rank difference) | 0.11 | 0.07 | [0.01;0.25] | 10784 | 1.00 | Student *t* (3,0,10) |
| sd(DSI) | 0.08 | 0.06 | [0.00;0.21] | 13191 | 1.00 | Student *t* (3,0,10) |
| cor(intercept, rank difference) | -0.21 | 0.46 | [-0.92;0.75] | 18094 | 1.00 | lkj_corr_cholesky(1) |
| cor(intercept, DSI) | -0.04 | 0.47 | [-0.87;0.84] | 27993 | 1.00 | lkj_corr_cholesky(1) |
| cor(rank difference, DSI) | 0.12 | 0.49 | [-0.84;0.91] | 24158 | 1.00 | lkj_corr_cholesky(1) |

Model 3 (RQ3): Exit phase presence (*N* =1129)

| *Fixed effects* | ***b*** | ***SE*** | **95% CrI** | ***Eff. N*** | $\hat{R}$ | ***Default Prior***[A] |
|---|---|---|---|---|---|---|
| Intercept | 3.48 | 0.38 | [2.78;4.30] | 34628 | 1.00 | Student *t* (3,0,10) |
| Species | | | | | | |
| Bonobo | *Reference* | | | | | |
| Chimpanzee | -1.55 | 0.39 | [-2.36;-0.81] | 41135 | 1.00 | lkj_corr_cholesky(1) |
| Age difference in years | -0.00 | 0.01 | [-0.02;0.02] | 41170 | 1.00 | lkj_corr_cholesky(1) |
| Sex | | | | | | |
| Different | *Reference* | | | | | |
| Same | 0.22 | 0.24 | [-0.26;0.69] | 56032 | 1.00 | lkj_corr_cholesky(1) |
| DSI | -0.71 | 0.32 | [-1.34;-0.07] | 28996 | 1.00 | lkj_corr_cholesky(1) |
| Rank difference | 0.04 | 0.41 | [-0.77;0.83] | 28061 | 1.00 | lkj_corr_cholesky(1) |
| DSI * Species | 0.87 | 0.39 | [0.12;1.67] | 27899 | 1.00 | lkj_corr_cholesky(1) |
| Rank difference * Species | -0.26 | 0.44 | [-1.11;0.63] | 26776 | 1.00 | lkj_corr_cholesky(1) |
| | | | | | | |
| *Random effects* | | | | | | |
| Initiator | | | | | | |
| sd(intercept) | 0.27 | 0.19 | [0.01;0.69] | 11387 | 1.00 | Student *t* (3,0,10) |
| sd(rank difference) | 0.37 | 0.23 | [0.02;0.88] | 9757 | 1.00 | Student *t* (3,0,10) |
| sd(DSI) | 0.49 | 0.23 | [0.06;0.96] | 10870 | 1.00 | Student *t* (3,0,10) |
| cor(intercept, rank difference) | 0.24 | 0.47 | [-0.76;0.94] | 12608 | 1.00 | lkj_corr_cholesky(1) |
| cor(intercept, DSI) | -0.08 | 0.47 | [-0.88;0.81] | 10024 | 1.00 | lkj_corr_cholesky(1) |
| cor(rank difference, DSI) | -0.35 | 0.45 | [-0.95;0.69] | 10095 | 1.00 | lkj_corr_cholesky(1) |
| Partner | | | | | | |
| sd(intercept) | 0.21 | 0.16 | [0.01;0.59] | 16264 | 1.00 | Student *t* (3,0,10) |
| sd(rank difference) | 0.18 | 0.14 | [0.01;0.53] | 20026 | 1.00 | Student *t* (3,0,10) |
| sd(DSI) | 0.41 | 0.24 | [0.02;0.93] | 8350 | 1.00 | Student *t* (3,0,10) |
| cor(intercept, rank difference) | -0.03 | 0.50 | [-0.89;0.87] | 39875 | 1.00 | lkj_corr_cholesky(1) |
| cor(intercept, DSI) | 0.09 | 0.49 | [-0.84;0.90] | 15120 | 1.00 | lkj_corr_cholesky(1) |

| | | | | | | |
|---|---|---|---|---|---|---|
| cor(rank difference, DSI) | -0.04 | 0.50 | [-0.89;0.86] | 14986 | 1.00 | lkj_corr_cholesky(1) |

| Model 4 (RQ3): Exit phase duration (*N* =1002) | | | | | | |
|---|---|---|---|---|---|---|
| *Fixed effects* | ***b*** | ***SE*** | **95% CrI** | ***Eff. N*** | $\hat{R}$ | ***Default Prior***[A] |
| Intercept | 2.26 | 0.08 | [2.10;2.42] | 26187 | 1.00 | Student *t* (3,2,10) |
| Species | | | | | | |
| Bonobo | *Reference* | | | | | |
| Chimpanzee | -0.09 | 0.09 | [-0.27;0.10] | 23089 | 1.00 | lkj_corr_cholesky(1) |
| Age difference in years | 0.00 | 0.00 | [-0.00;0.01] | 23569 | 1.00 | lkj_corr_cholesky(1) |
| Sex | | | | | | |
| Different | *Reference* | | | | | |
| Same | 0.00 | 0.07 | [-0.14;0.14] | 34499 | 1.00 | lkj_corr_cholesky(1) |
| DSI | -0.22 | 0.08 | [-0.39;-0.06] | 18948 | 1.00 | lkj_corr_cholesky(1) |
| Rank difference | -0.17 | 0.09 | [-0.35;0.01] | 17983 | 1.00 | lkj_corr_cholesky(1) |
| DSI * Species | 0.28 | 0.11 | [0.07;0.50] | 18088 | 1.00 | lkj_corr_cholesky(1) |
| Rank difference * Species | 0.15 | 0.11 | [-0.06;0.37] | 16719 | 1.00 | lkj_corr_cholesky(1) |
| | | | | | | |
| *Random effects* | | | | | | |
| Initiator | | | | | | |
| sd(intercept) | 0.09 | 0.06 | [0.00;0.22] | 7871 | 1.00 | Student *t* (3,0,10) |
| sd(rank difference) | 0.07 | 0.05 | [0.00;0.20] | 11223 | 1.00 | Student *t* (3,0,10) |
| sd(DSI) | 0.17 | 0.08 | [0.02;0.33] | 5741 | 1.00 | Student *t* (3,0,10) |
| cor(intercept, rank difference) | 0.06 | 0.50 | [-0.86;0.89] | 17225 | 1.00 | lkj_corr_cholesky(1) |
| cor(intercept, DSI) | -0.02 | 0.47 | [-0.86;0.84] | 6479 | 1.00 | lkj_corr_cholesky(1) |
| cor(rank difference, DSI) | -0.20 | 0.48 | [-0.92;0.79] | 7024 | 1.00 | lkj_corr_cholesky(1) |
| Partner | | | | | | |
| sd(intercept) | 0.13 | 0.07 | [0.01;0.27] | 4999 | 1.00 | Student *t* (3,0,10) |
| sd(rank difference) | 0.12 | 0.08 | [0.01;0.29] | 6619 | 1.00 | Student *t* (3,0,10) |
| sd(DSI) | 0.11 | 0.07 | [0.01;0.25] | 7668 | 1.00 | Student *t* (3,0,10) |
| cor(intercept, rank difference) | -0.11 | 0.46 | [-0.88;0.81] | 10586 | 1.00 | lkj_corr_cholesky(1) |
| cor(intercept, DSI) | 0.33 | 0.45 | [-0.71;0.94] | 8065 | 1.00 | lkj_corr_cholesky(1) |
| cor(rank difference, DSI) | 0.11 | 0.49 | [-0.83;0.90] | 11673 | 1.00 | lkj_corr_cholesky(1) |

*Note.* All datasets used for the mixed models excluded observations of exit phases that were identified as "mutually intitiated" phases, due to the problem of determining an ID of an initiator and partner for random effects.

[A]Bürkner, P.C. (2017). Brms: An R Package for Bayesian Multilevel Models Using Stan. *Journal of Statistical Software*, 80(1), 1-28. https://doi.org/10.18637/jss.v080.i01.

**Abbreviations**: b= Estimated mean of the posterior distribution; SD= Standard deviation of the posterior distribution; Crl= Two-sided 95% Credible intervals based on quantiles; *R*ˆ=R hat value, provides information about the convergence of the MCMC algorithm - if larger than 1.1, chains have not converged and model is not accurate; Effective N= number of effective sample size (i.e., number of independent samples from the posterior distribution, which would be expected to give the same standard error of posterior mean as obtained from dependent samples returned by MCMC algorithms).

**Table S6**. Leave-one-out cross-validation comparing intercept-only vs. random slope models. If ELPD differences were not greater than twice the SE, more parsimonious models were preferred. Related to STAR methods.

| *Model* | *LOO IC* | *SE* | *ELPD difference* | *SE difference* | *Pareto k diagnostic* | *Divergent chains?* |
|---|---|---|---|---|---|---|
| **model 1: Dependent variable: Entry phase presence** | | | | | | |
| *Intercept only model: initiator/partner intercepts only* | 1225.6 | 36.6 | 3.8 | 4.2 | Good (<0.5) | No |
| *Random slope model: DSI and Rank slopes for partner and initiator* | 1217.9 | 37.1 | | | OK (<0.7) | No |
| **model 2: Dependent variable: Entry phase duration** | | | | | | |
| *Intercept only model: initiator/partner intercepts only* | 6114.6 | 84.7 | 5.2 | 3.9 | Good (<0.5) | No |
| *Random slope model: DSI and Rank slopes for partner and initiator* | 6104.3 | 84.8 | | | Good (<0.5) | No |
| **model 3: Dependent variable: Exit phase presence** | | | | | | |
| *Intercept only model: initiator/partner intercepts only* | 766.9 | 43.8 | 1.5 | 3.9 | Good (<0.5) | No |
| *Random slope model: DSI and Rank slopes for partner and initiator* | 764.0 | 44.2 | | | OK (<0.7) | No |
| **model 4: Dependent variable: Exit phase duration** | | | | | | |
| *Intercept only model: initiator/partner intercepts only* | 7217.6 | 76.1 | 4.6 | 3.2 | Good (<0.5) | No |
| *Random slope model: DSI and Rank slopes for partner and initiator* | 7208.5 | 76.0 | | | OK (<0.7) | No |
